# WRAP53 Is Essential for Cajal Body Formation and for Targeting the Survival of Motor Neuron Complex to Cajal Bodies

**DOI:** 10.1371/journal.pbio.1000521

**Published:** 2010-11-02

**Authors:** Salah Mahmoudi, Sofia Henriksson, Irene Weibrecht, Stephen Smith, Ola Söderberg, Staffan Strömblad, Klas G. Wiman, Marianne Farnebo

**Affiliations:** 1 Department of Oncology-Pathology, Cancer Centrum Karolinska, Karolinska Institutet, Stockholm, Sweden; 2 Department of Genetics and Pathology, Rudbeck Laboratory, University of Uppsala, Uppsala, Sweden; 3 Center for Biosciences, Department of Biosciences and Nutrition, Novum, Karolinska Institutet, Huddinge, Sweden; National Cancer Institute, United States of America

## Abstract

The WRAP53 gene gives rise to a p53 antisense transcript that regulates p53. This gene also encodes a protein that directs small Cajal body–specific RNAs to Cajal bodies. Cajal bodies are nuclear organelles involved in diverse functions such as processing ribonucleoproteins important for splicing. Here we identify the WRAP53 protein as an essential factor for Cajal body maintenance and for directing the survival of motor neuron (SMN) complex to Cajal bodies. By RNA interference and immunofluorescence we show that Cajal bodies collapse without WRAP53 and that new Cajal bodies cannot be formed. By immunoprecipitation we find that WRAP53 associates with the Cajal body marker coilin, the splicing regulatory protein SMN, and the nuclear import receptor importinβ, and that WRAP53 is essential for complex formation between SMN–coilin and SMN–importinβ. Furthermore, depletion of WRAP53 leads to accumulation of SMN in the cytoplasm and prevents the SMN complex from reaching Cajal bodies. Thus, WRAP53 mediates the interaction between SMN and associated proteins, which is important for nuclear targeting of SMN and the subsequent localization of the SMN complex to Cajal bodies. Moreover, we detect reduced WRAP53–SMN binding in patients with spinal muscular atrophy, which is the leading genetic cause of infant mortality worldwide, caused by mutations in *SMN1*. This suggests that loss of WRAP53-mediated SMN trafficking contributes to spinal muscular atrophy.

## Abstract 2

The WRAP53 protein regulates the formation and maintenance of Cajal bodies (nuclear sub-organelles), as well as directs the recruitment of nuclear factors to Cajal bodies.

## Author Summary

Cajal bodies, discovered more than 100 years ago by Santiago Ramón y Cajal, are sub-organelles found in the nucleus of proliferative cells and neurons. They have been implicated in a variety of nuclear functions including ribonucleoprotein maturation, spliceosome formation, histone mRNA processing, RNA polymerase assembly, telomerase biogenesis, and histone gene transcription. Concentrating relevant molecules within Cajal bodies may serve to increase the efficiency of specific nuclear functions. Here we identify the WRAP53 protein as an essential factor for Cajal body maintenance and for directing the splicing regulatory protein “survival of motor neuron” (SMN) complex to Cajal bodies. We show that WRAP53 is a constitutive component of Cajal bodies, and that knockdown of WRAP53 disrupts existing Cajal bodies and prevents formation of new Cajal bodies. Mechanistically, we find that WRAP53 recruits the SMN complex from the cytoplasm to Cajal bodies by mediating interactions between SMN, importinβ, and coilin. Finally, we report deficient WRAP53–SMN binding in patients with spinal muscular atrophy, suggesting a role in this pathology. This study not only reveals new functions of the WRAP53 protein, but also increases our understanding of the molecular mechanism behind Cajal body formation and recruitment of factors to Cajal bodies.

## Introduction

We previously discovered *WRAP53* as an antisense gene to the p53 tumor suppressor gene [1]. *WRAP53* gives rise to a regulatory antisense transcript with a critical role for p53 function [1] and was recently approved as the official name of this gene (for “WD40 encoding RNA antisense to p53”; also denoted TCAB1 or WDR79). This gene also encodes a protein that directs small Cajal body–specific RNAs (scaRNAs), including the telomerase RNA, to Cajal bodies [2],[3]. Cajal bodies are nuclear organelles containing factors involved in ribonucleoprotein (RNP) maturation, spliceosome formation, histone mRNA processing, RNA polymerase assembly, telomerase biogenesis, and histone gene transcription [4]–[6]. The Cajal body was discovered more than 100 years ago by Santiago Ramón y Cajal, as a spherical structure often located in close proximity to the nucleolus (formerly called “nucleolar accessory body” or “coiled body”). Cajal bodies are dynamic structures that move within the nucleoplasm, move to and from nucleoli, join each other to form larger structures, and separate from larger into smaller bodies [7]. Nuclei contain 0–10 Cajal bodies, depending on cell cycle stage and cell type. Although Cajal bodies per se are not essential for cell survival, defects in Cajal body formation have been linked to impaired cell proliferation and splicing rates [8]–[10]. The reason why cells survive without Cajal bodies even though many processes in this organelle are essential for survival is probably that these processes can also occur in the nucleoplasm in the absence of Cajal bodies [11]. Thus, collecting enzymes and substrates in Cajal bodies may rather be a way to increase the efficiency of these processes by concentrating all factors at one site.

Cajal bodies are molecularly defined by the presence of the marker protein coilin. Coilin is essential for Cajal body integrity and function, and loss of coilin disrupts Cajal bodies. It has been proposed that coilin, upon oligomerization, provides a scaffold for the assembly of the different types of Cajal body components [12],[13] and that interaction with coilin mediates recruitment of proteins to Cajal bodies [14]. Formation of Cajal bodies also depends on spliceosomal small nuclear RNPs (snRNPs) that are rate-limiting factors for the assembly of additional Cajal bodies [10],[15]. Proteins involved in snRNP biogenesis, such as the survival of motor neuron (SMN) protein, are also important but not essential for Cajal body structure [10].

The SMN protein is part of a large complex essential for the assembly of snRNPs in the cytoplasm [16]. The SMN complex enables nuclear import of the snRNPs by binding to the nuclear import receptor importinβ [17],[18] and further transports the snRNPs to Cajal bodies for additional modification and maturation. Interaction between SMN and importinβ is required for SMN nuclear import, while SMN–coilin interaction is believed to mediate SMN complex localization to Cajal bodies [14]. Reduced levels of SMN due to mutations or deletions of the *SMN1* gene cause the common neurodegenerative disorder spinal muscular atrophy (SMA), the leading genetic cause of infant mortality worldwide, which affects approximately one in 6,000 infants. A second copy of the *SMN1* gene, *SMN2*, partially compensates for *SMN1* loss. However, because of a single nucleotide change, most *SMN2* transcripts lack exon 7, resulting in the production of the C-terminally truncated and unstable protein SMNΔC15 [19],[20]. The reason why the motor neurons in the spinal cord are selectively degenerated in SMN deficiency is still unknown. The clinical severity of this disease is correlated with low copy number of *SMN2* and reduced number of nuclear structures containing the SMN protein (encoded by *SMN2*) [21]–[23]. The latter suggests that targeting defects of SMN to nuclear structures contribute to SMA type I. In the present study, we have identified and characterized WRAP53 as a new critical player in Cajal body formation and for recruiting the SMN complex to Cajal bodies by mediating interactions between SMN, importinβ, and coilin. Moreover, WRAP53 and SMN association is disrupted in SMA patients, suggesting a role of WRAP53 in SMA pathogenesis.

## Results

### WRAP53 Is an Essential Component for Cajal Body Formation

**Figure 1:**
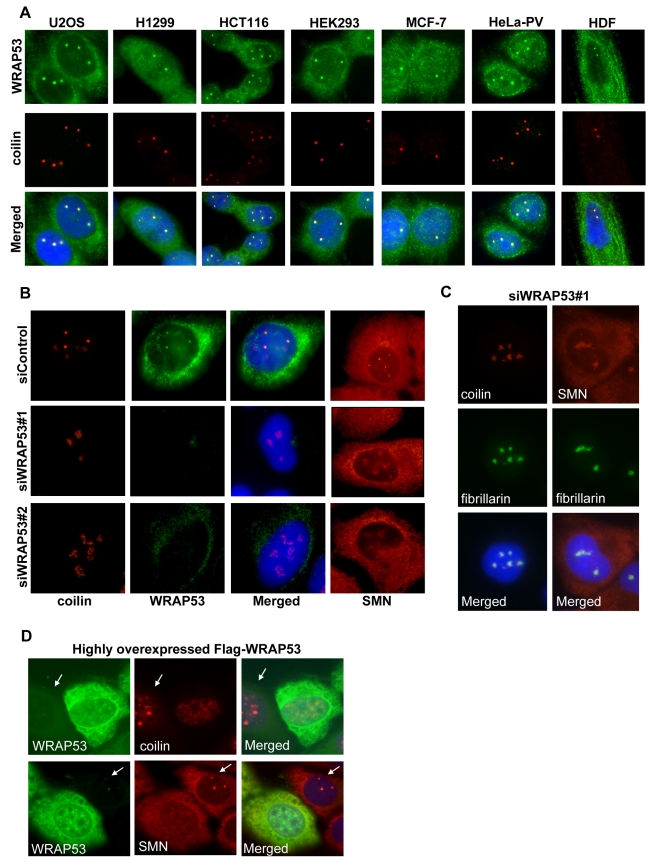
Aberrant expression of WRAP53 disrupts Cajal bodies and mislocalizes coilin and SMN. (A) IF staining of endogenous WRAP53 and the Cajal body marker coilin in different cell types. Nuclei were stained with DAPI in all IF experiments. (B) IF staining of coilin, WRAP53, and SMN in U2OS cells treated with the indicated siRNA oligos for 48 h. (C) U2OS cells treated with siWRAP53#1 for 48 h stained for either coilin or SMN together with the nucleolar marker fibrillarin. (D) U2OS cells transiently transfected with Flag-tagged full-length WRAP53 for 16 h and stained for WRAP53, coilin, and SMN. Arrows indicate untransfected cells used as controls.

The WRAP53 protein has been found highly enriched in nuclear Cajal bodies in HeLa cells [2],[3]. To further examine the presence of WRAP53 in Cajal bodies, a panel of cancer cell lines and primary cells including U2OS, H1299, HCT116, HEK293, MCF-7, HeLa-PV, and HDF were stained using a polyclonal antibody against WRAP53 and a monoclonal antibody against the Cajal body marker coilin. WRAP53 localized to Cajal bodies in all cell types analyzed (Figure 1A). Importantly, complete overlap between WRAP53 and coilin was observed in 100% of Cajal bodies in all cells (*n*>300), clearly indicating that WRAP53 is a constitutive component of Cajal bodies (Figure 1A).

To investigate whether WRAP53 plays a role in the formation or maintenance of Cajal bodies, WRAP53 was depleted in U2OS and HeLa cells, and the effects on Cajal bodies, i.e., coilin, was analyzed by immunoflourescence (IF) microscopy and Western blotting (WB). Two different small interfering RNA (siRNA) oligos targeting WRAP53 were used (siWRAP53#1 and siWRAP53#2), both knocking down WRAP53 mRNA with 90% efficiency (Figure S1A and S1B). In control cells, treated with a scramble siRNA with no homology to any gene (siControl), coilin displayed the characteristic Cajal body localization and co-localized with WRAP53 in all Cajal bodies (Figures 1B and S1C). A weak staining of coilin was also seen in nucleoli, consistent with previous findings that coilin transits through the nucleolus during the normal life cycle of the protein [13]. Strikingly, no Cajal bodies were found in WRAP53-depleted cells (Figures 1B and S1C). Instead, coilin accumulated in the nucleoli. Other Cajal body proteins, such as SMN, also showed absence of Cajal body accumulation and increased nucleolar staining in WRAP53-depleted cells (Figures 1B and S1C). Staining with the nucleolar marker fibrillarin confirmed nucleolar accumulation of coilin and SMN upon WRAP53 depletion (Figure 1C). Thus, WRAP53 is required for Cajal body maintenance. WRAP53-depleted cells were also analyzed for changes in other nuclear structures, such as nucleoli (fibrillarin), gems (SMN), and promyelocytic leukemia (PML) bodies. No effects on these structures were observed (Figure S1D), demonstrating that WRAP53 is an essential component for Cajal bodies but is not essential for other nuclear structures.

Loss of Cajal bodies was strictly associated with the degree of WRAP53 knockdown, where complete knockdown of WRAP53 led to the disappearance of all Cajal bodies, and cells still expressing low levels of nuclear WRAP53 showed Cajal body staining (Figure S1C). We also knocked down coilin and SMN in U2OS and HeLa cells. Depletion of coilin resulted in the disappearance of all Cajal bodies, leaving WRAP53 and SMN dispersed throughout the nucleoplasm (Figure S1E). Depletion of SMN significantly reduced the number and size of Cajal bodies, but some cells still had Cajal bodies left. Both WRAP53 and coilin were present in the remaining Cajal bodies and accumulated in nucleoli (Figure S1F). Thus, WRAP53 and coilin are essential for Cajal body structure, whereas SMN is not.

We next examined the effects on Cajal bodies in cells overexpressing WRAP53. Flag-tagged WRAP53 expressed at lower levels showed Cajal body accumulation (Figure S2A). In contrast, Flag-WRAP53 expressed at higher levels gave rise to a different nuclear expression pattern, with a more even distribution throughout the nucleoplasm (Figures 1D and S2A). Interestingly, no Cajal bodies were detected in these cells, and coilin and SMN were, like WRAP53, distributed throughout the nucleoplasm. Similar phenomena were observed using enhanced green fluorescent protein (EGFP)–tagged WRAP53 (Figure S2B). WB analysis of WRAP53 knockdown and WRAP53-overexpressing cells showed no difference in coilin or SMN protein levels (Figures S1B and S2C), and immunostaining of WRAP53-overexpressing cells showed no change in other nuclear structures, including PML bodies (Figure S2D). Thus, aberrant overexpression of WRAP53 prevents Cajal body formation and causes significant mislocalization of the Cajal body proteins coilin and SMN to the nucleoplasm. This finding confirms the notion that WRAP53 is an essential component of Cajal body structure and that proper localization of WRAP53 is required for its role in Cajal body formation.

### WRAP53 Is Required for De Novo Formation of Cajal Bodies Induced by SMN Overexpression

**Figure 2:**
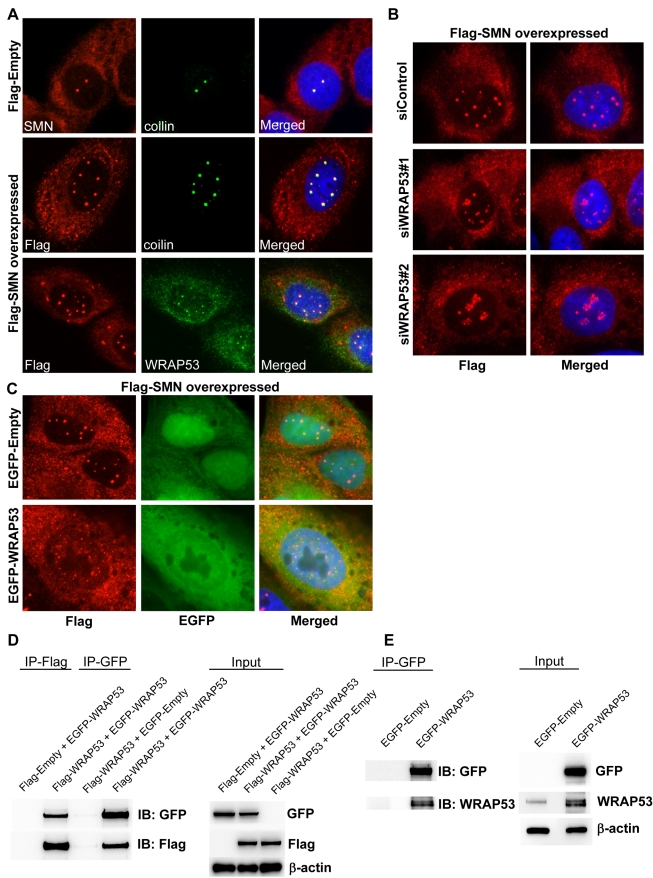
WRAP53 is required for formation of Cajal bodies induced by SMN overexpression, and it self-associates. (A) IF using SMN, Flag, coilin, and WRAP53 antibodies in U2OS cells transiently transfected with the indicated constructs for 16 h. (B) IF using Flag antibody in U2OS cells treated with siWRAP53 for 24 h, followed by transfection with Flag-tagged SMN for additional 24 h. (C) IF using Flag antibody in U2OS cells co-transfected with Flag-SMN and EGFP-Empty or with Flag-SMN and EGFP-WRAP53FL. (D) IP using Flag or GFP antibodies in U2OS cells transiently transfected with the indicated constructs for 24 h. (E) IP using GFP antibody in U2OS cells transiently transfected with the indicated constructs for 24 h. IB, immunoblotting.

snRNPs are known to be rate-limiting for Cajal body formation [8],[24]. The SMN complex transports snRNPs into the nucleus, and overexpressing the SMN protein induces formation of additional Cajal bodies. In light of this knowledge, we examined the influence of WRAP53 on de novo formation of Cajal bodies. Flag-tagged SMN was transiently transfected into U2OS cells, which increased the number of Cajal bodies per cell from 2–3 in control cells up to ten in Flag-SMN cells (Figure 2A). All Cajal bodies were positive for WRAP53 and coilin (Figure 2A). Cytosolic accumulations of SMN were observed in Flag-SMN cells; however, neither coilin nor WRAP53 were present in these structures (Figure 2A). Interestingly, both knockdown and aberrant overexpression of WRAP53 repressed generation of Cajal bodies induced by SMN overexpression (Figure 2B and 2C). Instead, Flag-SMN mislocalized to the nucleoli in WRAP53-depleted cells and to the nucleoplasm in WRAP53-overexpressing cells. These results show that WRAP53 is required for formation of new Cajal bodies induced by SMN overexpression, which further supports the idea that WRAP53 is essential for Cajal body assembly.

The finding that exogenous WRAP53 alters the localization of endogenous WRAP53, SMN, and coilin suggests a dominant negative effect of overexpressed WRAP53 that could be caused by WRAP53 self-interaction. Previous reports demonstrate such phenomena for coilin, where overexpressed coilin mislocalizes to nucleoli and disrupts Cajal bodies through dominant negative interference between exogenous and endogenous coilin [13]. To investigate if WRAP53 self-associates, U2OS cells were co-transfected with Flag-WRAP53 and EGFP-WRAP53 constructs. Immunoprecipitation (IP) with anti–green fluorescent protein (GFP) or anti-Flag antibodies showed that Flag-WRAP53 protein co-precipitated EGFP-WRAP53 and vice versa (Figure 2D). This indicates that exogenous WRAP53 self-associates in vivo. Furthermore, IP of EGFP-WRAP53 in U2OS cells co-precipitated endogenous WRAP53, whereas IP of EGFP alone did not (Figure 2E). This suggests that overexpressed WRAP53 interacts with endogenous WRAP53 in vivo, which also strengthens our hypothesis that overexpressed EGFP-WRAP53 or Flag-WRAP53 can cause mislocalization of endogenous WRAP53 by self-association.

### The WD40 Domain and C-Terminal Region of WRAP53 Mediates Interaction with Coilin and SMN and Targets WRAP53 to Cajal Bodies

**Figure 3:**
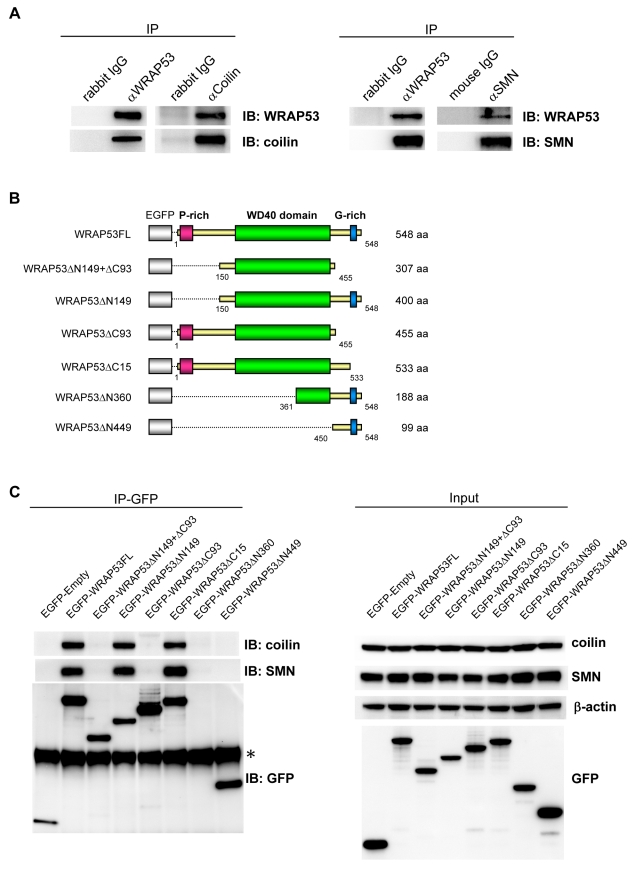
WRAP53 binds coilin and SMN via its WD40 domain and C-terminal region. (A) IP of endogenous WRAP53, coilin, and SMN from U2OS cells followed by immunoblotting (IB) with the indicated antibodies. Rabbit and mouse IgG were used as negative controls. (B) Schematic illustration of EGFP-tagged WRAP53 deletion constructs: WRAP53FL (full-length), WRAP53ΔN149+ΔC93 (contains aa 150–455), WRAP53ΔN149 (contains aa 150–584), WRAP53ΔC93 (contains aa 1–455), WRAP53ΔC15 (contains aa 1–533), WRAP53ΔN360 (contains aa 361–548), and WRAP53ΔN449 (contains aa 450–548). The EGFP protein has a molecular weight of approximately 27 kDa. (C) U2OS cells transfected with the indicated WRAP53 constructs for 16 h, followed by IP with GFP antibody. Asterisk indicates the heavy chain. The WRAP53ΔN360 product is 50 kDa in size and is thus covered by the heavy chain.

IP of endogenous WRAP53 furthermore revealed that WRAP53 associates with coilin and SMN (Figure 3A). Reciprocal IP of coilin and SMN verified the interactions with WRAP53. To assess which region of WRAP53 interacts with coilin and SMN, we generated and transiently overexpressed a series of EGFP-tagged WRAP53 deletion constructs in U2OS cells (Figure 3B). Each construct expressed a protein of the expected size, as demonstrated by immunoblotting using both GFP and WRAP53 antibodies (Figure 3C and data not shown). IP of EGFP-WRAP53 using GFP antibody showed that WRAP53 constructs containing the WD40 domain plus the C-terminal region containing amino acids (aa) 456–533 associated with coilin and SMN. Constructs lacking these two domains or only expressing one of them co-precipitated neither coilin nor SMN (Figure 3C). Hence, WRAP53 associates with both coilin and SMN, and the same sequence in WRAP53 is important for interaction with both these proteins.

**Figure 4:**
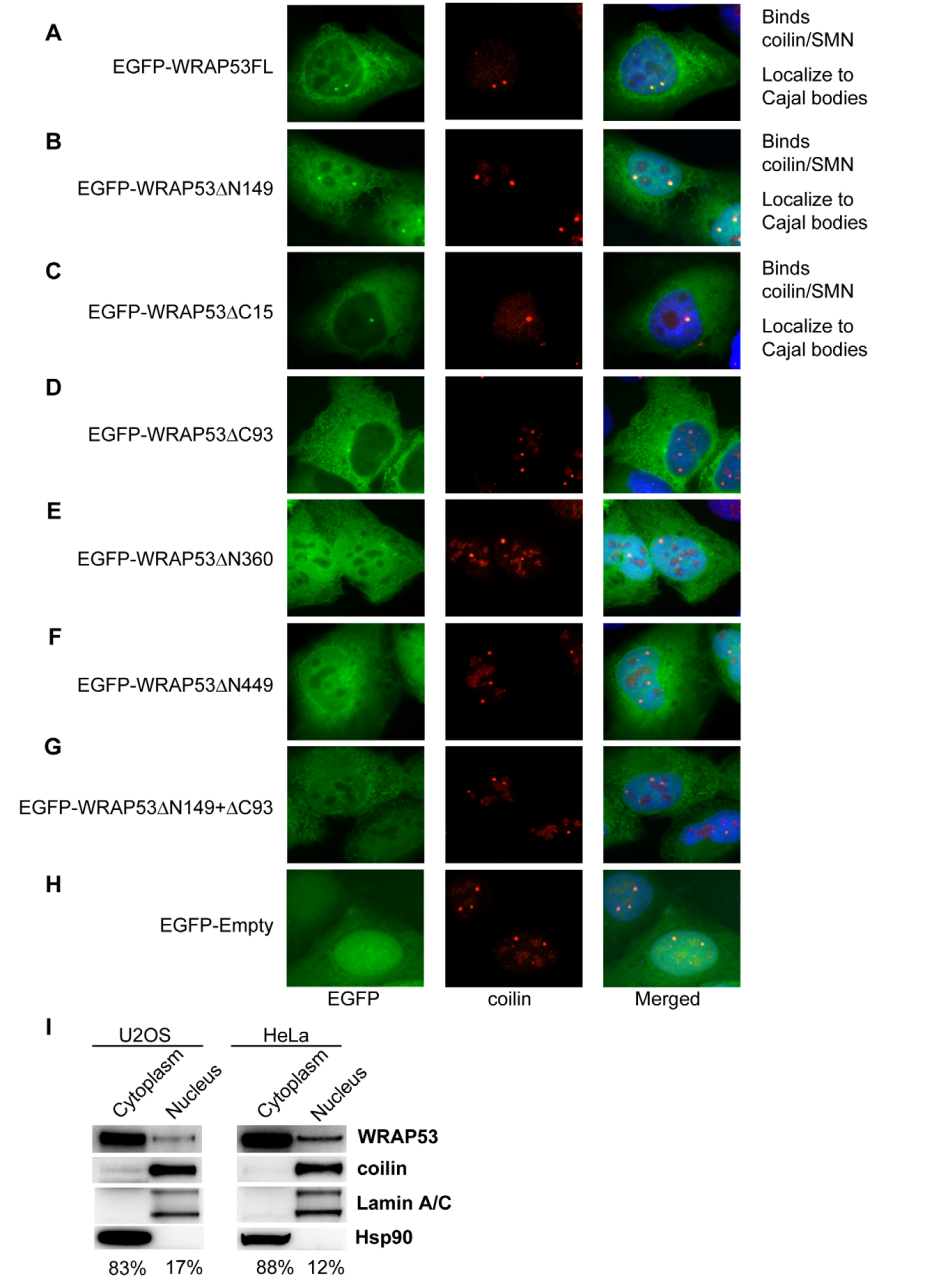
The WD40 domain and C-terminal region targets WRAP53 to Cajal bodies. (A–H) U2OS cells transiently transfected with the indicated EGFP-tagged constructs for 16 h and stained for coilin. (I) WB analysis of nuclear and cytoplasmic fractions from U2OS and HeLa cells. In all fractionations Lamin A/C and Hsp90 were used as nuclear and cytoplasmic markers, respectively. Densitometric quantifications of the different WRAP53 fractions compared to total levels of WRAP53 are indicated below the panels.

To investigate which region of WRAP53 mediates its localization to Cajal bodies, the panel of EGFP-WRAP53 deletion constructs was transiently transfected into U2OS cells, and protein localization was analyzed by IF. The cells were also stained for coilin to visualize Cajal bodies. Interestingly, only the WRAP53 constructs that bind coilin and SMN (EGFP-WRAP53FL, EGFP-WRAP53ΔN149, and EGFP-WRAP53ΔC15) accumulated in Cajal bodies (Figure 4A–4C). In contrast, the constructs unable to bind coilin or SMN failed to localize to Cajal bodies (Figure 4D–4H). This suggests that interaction with coilin and/or SMN is necessary for WRAP53 localization to Cajal bodies. No change in Cajal body number was observed in cells overexpressing the different WRAP53 constructs (data not shown). These observations were made in cells expressing low to moderate levels of WRAP53. In cells with high WRAP53 expression, nuclear mislocalization of WRAP53, SMN, and coilin was observed. Most likely this is due to sequestering of coilin and SMN in the nucleoplasm by EGFP-WRAP53, since high expression of WRAP53 deletion mutants unable to bind coilin did not mislocalize coilin/SMN and had no effects on Cajal body appearance (data not shown). Thus, the WD40 domain and the C-terminal region of WRAP53 target WRAP53 to Cajal bodies.

We also observed that all WRAP53 constructs showed cytoplasmic localization (Figure 4A–4G) and that WRAP53 constructs lacking the C-terminal region (EGFP-WRAP53ΔC93 and EGFP-WRAP53ΔC15) demonstrated a more pronounced cytoplasmic staining (Figure 4C and 4D). This was most apparent with the EGFP-WRAP53ΔC93 construct. In contrast, N-terminally deleted constructs exhibited the opposite distribution, i.e., a stronger nuclear staining (Figure 4B, 4E, and 4F), which was most apparent with the EGFP-WRAP53ΔN149 construct. These results indicate that the C- and N-terminal regions of WRAP53 contain elements important for the subcellular distribution of WRAP53.

Previous studies failed to detect any WRAP53 protein in the cytoplasm [2],[3]. To investigate this further we performed IF staining of endogenous WRAP53 with three different WRAP53 antibodies. Interestingly, all three antibodies show cytoplasmic localization of WRAP53, in addition to accumulation in Cajal bodies, using both methanol and paraformaldehyde fixation (Figures 1A, S3B, and S3C). A clear reduction in cytoplasmic and nuclear WRAP53 staining was observed after WRAP53 depletion, confirming the specificity of the WRAP53 staining in both compartments (Figure 1B). We also performed cell fractionation followed by WB of the WRAP53 protein. This confirmed that WRAP53 is present both in cytoplasmic and nuclear fractions, and quantification of the blots revealed that 83%–88% of the WRAP53 protein is localized to the cytoplasm (Figure 4I). Thus, endogenous and exogenous WRAP53 (Flag-WRAP53 and EGFP-WRAP53) show a clear cytoplasmic localization. We conclude that WRAP53 localizes to the cytoplasm in addition to nuclear Cajal bodies.

### WRAP53 Is Required for Coilin–SMN Complex Formation and Influences the Intracellular Distribution of SMN

**Figure 5:**
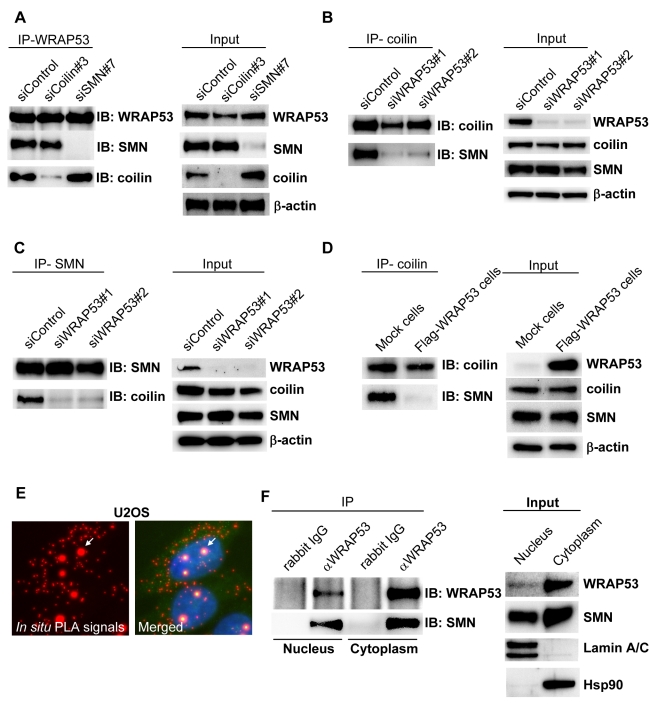
WRAP53 mediates coilin–SMN interaction and associates with SMN both in the cytoplasm and in the nucleus. (A–C) IP of endogenous WRAP53 (A), coilin (B), and SMN (C) from U2OS cells pretreated with the indicated siRNA oligos for 48 h. (D) IP of endogenous coilin from U2OS cells stably transfected with empty vector (Mock cells) or Flag-WRAP53 (Flag-WRAP53 cells). The Flag-WRAP53 cells express high levels of WRAP53 and show subsequent disruption of Cajal bodies (see Figure S4A). (E) In situ PLA detection of the interaction between WRAP53 and SMN in U2OS cells. Protein–protein interactions are visualized as small, distinct red spots. In Cajal bodies several in situ PLA signals are superimposed. Arrows indicate one Cajal body. (F) IP of endogenous WRAP53 from nuclear and cytoplasmic fractions of U2OS cells.

To understand the organization of interaction between WRAP53, SMN, and coilin, we separately knocked down each of these proteins, and then performed IP analysis. This showed that WRAP53 co-precipitates coilin in SMN-depleted cells and co-precipitates SMN in coilin-depleted cells (Figure 5A). Thus, WRAP53 interacts with coilin independently of SMN and with SMN independently of coilin. In contrast, IP of coilin and SMN in WRAP53-depleted cells showed a significant reduction in coilin–SMN interaction (Figure 5B and 5C). Loss of coilin–SMN complex formation was also found in U2OS cells stably overexpressing Flag-WRAP53 at high levels (Figure 5D) and displaying nucleoplasmic mislocalization of WRAP53 and coilin (Figure S4A). These results demonstrate that proper expression of WRAP53 is required for coilin–SMN complex formation in vivo.

Previous studies have described a direct binding between SMN and coilin [14], suggesting that WRAP53 is not required for the actual interaction between these proteins but rather brings them in close proximity to allow their interaction. Since WRAP53 localizes to the cytoplasm, we hypothesized that WRAP53 recruits SMN from the cytoplasm to nucleus, thus enabling interaction between SMN and coilin. Using in situ proximity ligation assay (in situ PLA) [25], we found that WRAP53 and SMN associate both in the cytoplasm and in Cajal bodies (Figures 5E and S4B). In situ PLA is a sensitive method that detects endogenous protein–protein interactions (visualized as red dots) in fixed cells and allows identification of the precise subcellular localization of the interaction. Cell fractionation followed by IP of endogenous WRAP53 furthermore confirmed that WRAP53 associates with SMN both in the cytoplasm and in the nucleus (Figure 5F). Thus, WRAP53 and SMN interact both in the cytoplasm and nucleus.

**Figure 6:**
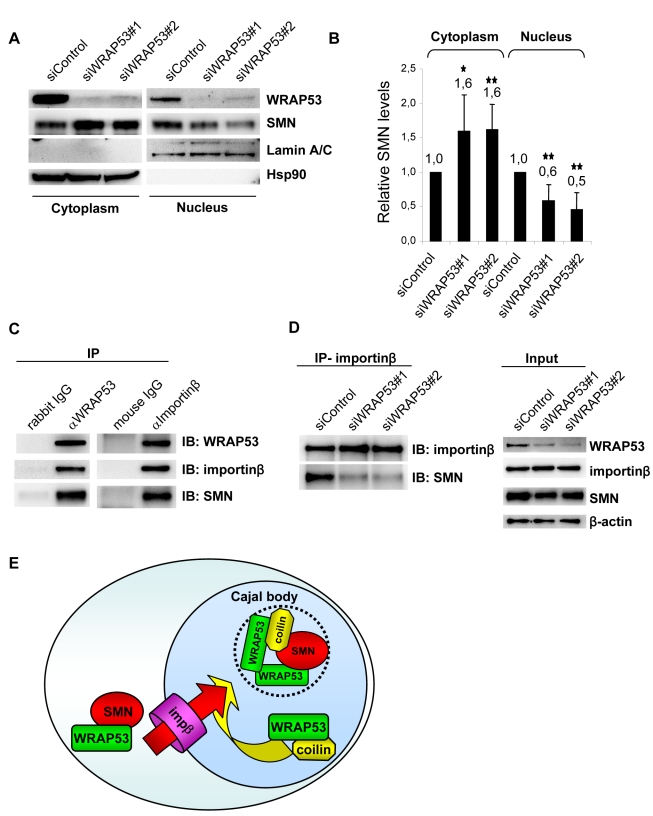
WRAP53 regulates SMN distribution and mediates SMN–importinβ interaction. (A) WB analysis of SMN levels in fractionated U2OS cells treated with the indicated siRNA oligos. (B) Densitometric quantifications of SMN levels in fractionated U2OS cells treated with the siRNAs shown in (A). Levels of SMN have been normalized against the internal fractionation control. The graph shows means of five independent experiments; error bars represent standard error; one asterisk indicates *p<*0.05, two asterisks indicate *p<*0.01, according to the Student's *t* test. (C) IP of endogenous WRAP53 and importinβ from U2OS cells. Rabbit and mouse IgG were used as negative controls. (D) IP of endogenous importinβ from U2OS cells pretreated with the indicated siRNA oligos for 48 h. (E) Hypothetical model of WRAP53-mediated SMN nuclear import and Cajal body formation. WRAP53 recruits SMN from the cytoplasm by facilitating SMN–importinβ (impβ) interaction and further mediates SMN–coilin interaction in the nucleus, which enables SMN to reach Cajal bodies. WRAP53 self-association could be the event that brings coilin and SMN together and facilitates Cajal body formation

We next analyzed whether knockdown of WRAP53 affects the cytoplasmic and nuclear distribution of SMN. Interestingly, a significant increase of SMN was found in the cytoplasm in WRAP53-depleted cells, which coincided with a decrease of SMN in the nucleus (Figure 6A). This was demonstrated by cell fractionation and WB, and quantification of the Western blots showed a 60% increase of SMN in the cytoplasm and 40%–50% decrease of SMN in the nucleus upon WRAP53 knockdown (Figure 6B). Knockdown of coilin did not affect the intracellular distribution of SMN (Figure S5A), indicating that the function of WRAP53 in Cajal body assembly is not underlying the changed SMN distribution. The subcellular distribution of WRAP53 was not affected by SMN depletion, indicating that WRAP53 controls SMN but not vice versa (Figure S5A). Thus, knockdown of WRAP53 results in cytoplasmic accumulation and nuclear decrease of SMN, supporting our hypothesis that WRAP53 is involved in the recruitment of SMN from the cytoplasm to the nucleus.

Interaction with the nuclear import receptor importinβ is required for SMN nuclear import [18]. Co-IPs of importinβ and WRAP53 showed that the two proteins interact (Figure 6C). To test whether WRAP53 influences interaction between SMN and importinβ, cells were depleted for WRAP53 and immunoprecipitates of importinβ were assayed for SMN. Importinβ efficiently associated with SMN in siControl-treated cells (Figure 6D). In contrast, a significantly smaller amount of SMN protein was co-precipitated with importinβ antibody in siWRAP53-treated cells (Figure 6D), showing that WRAP53 is important for SMN–importinβ association. Thus, knockdown of WRAP53 reduces importinβ and SMN binding, diminishes the nuclear localization of SMN, and causes SMN to accumulate in the cytoplasm. It appears as if WRAP53 recruits SMN from the cytoplasm to the nucleus by facilitating SMN–importinβ interaction and subsequently mediates interaction between SMN and coilin in the nucleus by bringing the proteins in close proximity. This may either occur within an already existing Cajal body or catalyze the formation of a new Cajal body (Figure 6E).

### WRAP53 Is Required for Localization of the SMN Complex and snRNP to Cajal Bodies

We next examined whether WRAP53 is required for directing other components of the SMN complex to Cajal bodies, and Gemin3 was chosen as a representative component. IP analysis showed that WRAP53 and Gemin3 indeed associate (Figure S5B). Depletion of SMN disrupted this interaction, showing that WRAP53 and Gemin3 interact through SMN (Figure S5B). Knockdown of WRAP53 did not affect SMN–Gemin3 binding (Figure S5C). Like SMN, Gemin3 also showed absence of Cajal body accumulation in WRAP53-depleted or -overexpressed cells, but were still localized in gems (). In WRAP53-depleted cells, Gemin3 localized to the nucleoplasm, to gems, and partially to nucleoli (Figure S5D and S5F). Altered localization of Gemin2 was also observed in WRAP53-depleted cells (Figure S5F). Like SMN, Gemin2 localized to nucleoli and gems upon WRAP53 knockdown. Taken together, WRAP53 is important for localizing the entire SMN complex to Cajal bodies but not to gems.

We also analyzed whether WRAP53 targets snRNPs to Cajal bodies. snRNPs consist of Sm proteins in complex with small nuclear RNAs and are carried by the SMN complex from the cytoplasm to Cajal bodies, where final maturation of the snRNPs takes place. In control cells the Sm proteins were enriched in Cajal bodies and distributed throughout the nucleus in speckles (Figure S6A). In WRAP53-depleted cells, no Sm accumulation in Cajal bodies was observed, but Sm was still present in speckles (Figure S6A). Similary, upon SMN knockdown, Sm did not localize to Cajal bodies but to speckles (data not shown). Thus, WRAP53 is required for Sm localization to Cajal bodies. Knockdown of WRAP53 did not affect SMN–Sm binding nor the cytoplasmic and nuclear distribution of Sm (Figures S5C and S6B), indicating that loss of Sm accumulation in Cajal bodies is not caused by snRNP assembly defects or altered intracellular distribution of Sm.

### Interaction of WRAP53 and SMN Is Disrupted in SMA Type I Patients

**Figure 7:**
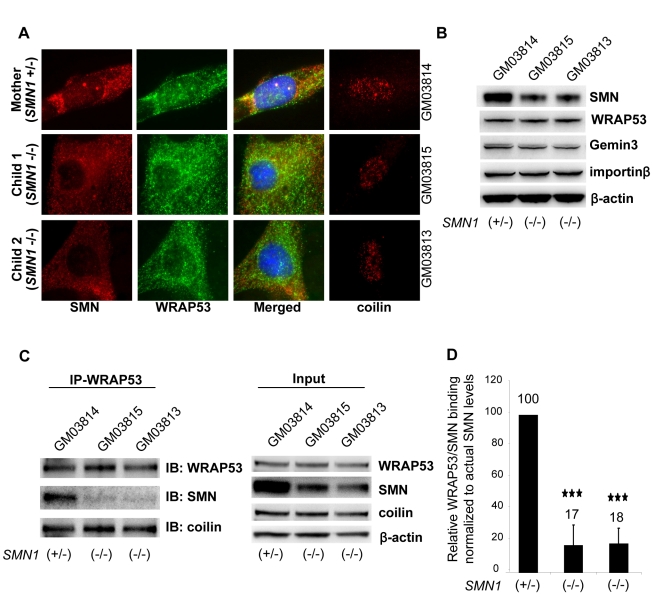
Interaction of WRAP53 and SMN is disrupted in SMA type I patients. (A) IF staining of SMN, WRAP53, and coilin in fibroblasts derived from the unaffected mother (GM03814) and her two children with SMA type I (GM03815 and GM03813). (B) WB analysis of WRAP53, SMN, Gemin3, and importinβ levels in fibroblasts from the same individuals as described in (A). β-actin was used as loading control. (C) IP of endogenous WRAP53 from the same fibroblasts described in (A). (D) Densitometric quantifications of the relative WRAP53–SMN interaction in the fibroblasts described in (A). Levels of SMN co-immunoprecipitated with WRAP53 antibody have been normalized to the levels of immunoprecipitated WRAP53, as well as to the relative amount of SMN in the cells. The latter was calculated by dividing SMN levels in the input with β-actin levels in the input. The graph shows means of three independent experiments; error bars represent standard error, three asterisks indicate *p<*0.001 (Student's *t* test).

The most severe form SMA, SMA type I, correlates with a reduced number of SMN-containing nuclear bodies [21]–[23]. The role of WRAP53 in Cajal body formation and nuclear localization of SMN encouraged us to investigate the interplay of WRAP53 and SMN in vivo in SMA disease. We first analyzed SMN localization in nuclear bodies in fibroblasts derived from an unaffected mother (GM03814, serving as control) and her two children with SMA type I (GM03815 and GM03813). Co-staining of SMN and coilin showed that SMN accumulated in nuclear bodies (gems and Cajal bodies) in 67% of control fibroblasts (GM03814), compared to only 13% (GM03815) and 16% (GM03813) of SMA fibroblasts. Cajal bodies (coilin accumulation) were detected in 43% of control fibroblasts (GM03814), but in only 25% (GM03815) and 15% (GM03813) of the SMA fibroblasts. WRAP53 was present in all Cajal bodies. The absence of SMN in nuclear bodies coincides with lack of Cajal bodies in the same cells (Figure 7A). Thus, both gem and Cajal body number are decreased in SMA fibroblasts. This observation could not be explained by the difference in WRAP53 levels in SMA fibroblasts compared to control (Figure 7B). However, WRAP53 showed reduced binding to SMN protein in the SMA fibroblasts (Figure 7C). To investigate whether the lack of binding between WRAP53 and SMN was just a reflection of decreased levels of SMN in the SMA fibroblasts, or whether WRAP53 binding to the SMN protein derived from the *SMN2* allele is in fact weaker, we quantified the relative amount of SMN that interacts with WRAP53 in SMA patients and in normal cells. This revealed a lack of binding between SMN and WRAP53 in cells from SMA patients that cannot be explained by the lower amounts of SMN, and that the relative binding between WRAP53 and SMN in these cells was reduced by 83% (Figure 7D). We did not observe altered binding between WRAP53 and coilin in the SMA cells (Figure 7C), demonstrating that the lost interaction between WRAP53 and SMN is specific and not a secondary effect of disrupted WRAP53–coilin interaction. We thus conclude that the interaction between WRAP53 and SMN is disrupted in SMA type I patients, which further relates to a failure of SMN accumulation in nuclear bodies.

## Discussion

Here we identify WRAP53 as an essential factor for Cajal body maintenance and for directing the SMN complex to Cajal bodies. We show that WRAP53 is a constitutive component of Cajal bodies that overlaps coilin in 100% of Cajal bodies in a variety of cell lines. Knockdown of WRAP53 disrupts Cajal bodies, prevents formation of new Cajal bodies, and relocates Cajal body proteins coilin and SMN from Cajal bodies to nucleoli. WRAP53 seems specifically important for Cajal body integrity, since depletion of WRAP53 does not affect gems or other nuclear structures, including nucleoli and PML bodies. We show that WRAP53 separately associates with coilin and SMN and is required for their complex formation. Previous studies demonstrated a direct interaction between SMN and coilin, suggesting that WRAP53 is not important for their binding but rather mediates interaction by bringing the proteins in close proximity. This may either occur within an already existing Cajal body or result in the formation of a new Cajal body. Importantly, WRAP53's role in Cajal body formation goes beyond bringing SMN and coilin together, since knockdown of SMN does not abolish all Cajal body structures. Residual Cajal bodies containing WRAP53 and coilin still remain. Moreover, in HeLa-PV cells, WRAP53 and coilin localize to all Cajal bodies and SMN to only 40% of them (*n = *100) (data not shown). These observations, together with the fact that knockdown of WRAP53 or coilin disrupts all Cajal body structures, point to a more general function of WRAP53 in assisting coilin as a scaffold protein in Cajal body formation.

Cajal bodies have been suggested to have separate compartments containing snRNP, snoRNP/scaRNP, or basal transcription factors [10]. Depletion of proteins involved in snRNP maturation, such as SMN, TGS1, and PHAX, disrupts canonical Cajal bodies containing snRNP, whereas residual Cajal bodies lacking snRNPs but containing coilin and snoRNP/scaRNP components still remain. Without WRAP53, both canonical and residual Cajal bodies collapse, suggesting that WRAP53 is important for processes in addition to snRNP maturation. WRAP53 has been shown to be essential for scaRNA, including telomerase RNA, localization to Cajal bodies [2],[3], which could account for some of the observed defects in Cajal body formation upon WRAP53 perturbation.

We also observe that high overexpression of WRAP53 disassembles Cajal bodies and results in nucleoplasmic mislocalization of WRAP53, coilin, and SMN. This indicates that overexpressed WRAP53 has a dominant negative effect on WRAP53 function and that exogenous and endogenous WRAP53 may compete for factors important for WRAP53 localization to Cajal bodies and Cajal body formation. The fact that endogenous WRAP53 co-precipitates with exogenous WRAP53 indicates that WRAP53 can self-associate, which can also explain the observed effect. Indeed, self-oligomerization appears to be a general feature of nuclear body marker proteins including coilin, SMN, and PML [26]–[28], which is consistent with our findings that WRAP53 is a signature protein for Cajal bodies. In line with this notion, overexpression of coilin also disrupts Cajal bodies and results in coilin mislocalization [13]. Hypothetically, WRAP53 self-association could be the event that brings coilin and SMN together and facilitates Cajal body formation.

The effects on Cajal bodies of depletion and overexpression of WRAP53 are highly similar to those of loss or overexpression of coilin. Coilin mutants have been described in human, mouse [8], *Arabidopsis* [29], and *Drosophila* [30]. In all of these species, loss of coilin produces defects in Cajal body formation. Overexpression of coilin, on the other hand, produces slightly different effects in the different organisms. In *Drosophila* and *Arabidopsis*, enhanced coilin levels result in normal Cajal body formation or formation of larger Cajal bodies, whereas overexpression of coilin in mice and human cells disrupts Cajal bodies, as previously described. It would be interesting to investigate the effects of WRAP53 depletion and overexpression in other organisms as well.

Deletion mapping of the WRAP53 protein demonstrates that the WD40 domain plus C-terminal aa 456–533 are required for interaction with coilin and SMN. The same domains also target WRAP53 to Cajal bodies, suggesting that interaction with coilin and/or SMN mediates this localization. The apparent lack of SMN in a fraction of WRAP53- and coilin-associated Cajal bodies, however, indicates that coilin is the important factor for WRAP53's nuclear localization. However, we cannot exclude that yet unidentified interaction partners of WRAP53, binding via the same regions, may also be important for localization of WRAP53 to Cajal bodies.

Interestingly, we found that WRAP53 also localizes to the cytoplasm, which is in contrast to two previous reports [2],[3]. Our conclusion is based on the following findings: (1) three different WRAP53 antibodies all demonstrate WRAP53 localization to the cytoplasm, (2) overexpressed Flag- and EGFP-tagged WRAP53 shows cytoplasmic localization, (3) WRAP53 knockdown efficiently depletes WRAP53 staining in both locations, (4) cytoplasmic localization of WRAP53 is shown in seven different cell lines and with different fixation protocols, and (5) cell fractionation followed by WB confirms presence of WRAP53 in the cytoplasm.

We find that WRAP53 associates with SMN both in the cytoplasm and in the nucleus and influences the intracellular distribution of SMN. Knockdown of WRAP53 results in SMN accumulation in the cytoplasm and decreased SMN in the nucleus. Thus, cytoplasmic WRAP53 seems to recruit SMN from the cytoplasm to the nucleus. This role of WRAP53 is separate from WRAP53's function in Cajal body formation, since knockdown of coilin does not affect the intracellular distribution of SMN. Moreover, knockdown of WRAP53 abrogates interaction between SMN and the nuclear pore receptor importinβ, which could explain the skewed intracellular distribution of SMN observed in WRAP53-depleted cells. Nuclear localization of SMN has been shown to depend on SMN–importinβ complex formation but also on other factors such as proper snRNP assembly [18]. Importantly, WRAP53 depletion does not affect the interaction between SMN, Gemin3, and Sm. This suggests that WRAP53 does not promote SMN complex formation nor snRNP assembly but rather is important for SMN-associated interactions occurring after these events. It appears as if WRAP53 recruits the SMN complex from the cytoplasm by facilitating SMN–importinβ complex formation and further mediates interaction between SMN and coilin in the nucleus by bringing the proteins in close proximity. Our finding that Sm is not retained in the cytoplasm upon WRAP53 depletion is in line with several previous studies where no cytosolic snRNP accumulations were observed upon silencing of SMN, Gemin3, or Gemin4 [10],[31],[32]. The very stable nature of mature snRNPs [33] and additional import mechanisms for snRNPs independent of SMN–importinβ interaction have been suggested as plausible reasons.

We also find that the interaction between SMN and WRAP53 is disrupted in fibroblasts from SMA patients and that this correlates with a reduced number of SMN foci in the nuclei of these cells. The reason for the loss of binding between WRAP53 and SMN in SMA is currently unknown but opens the possibility that WRAP53 could contribute to SMN dysfunction in SMA. In summary, we have demonstrated two important functions of the WRAP53 protein. First, it acts as a critical scaffold protein for Cajal body formation, along with coilin. Second, it recruits the SMN complex from the cytoplasm to nuclear Cajal bodies by mediating interaction between SMN, importinβ, and coilin. This discovery not only reveals new functions of the WRAP53 protein but also increases our understanding of the molecular mechanism behind Cajal body formation and recruitment of factors to Cajal bodies.

## Material and Methods

### Culture Conditions

U2OS, H1299, HCT116, HEK293, MCF-7, HeLa-PV, HeLa-ATCC, and HDF cells were maintained in Dulbecco's modified medium supplemented with 10% fetal bovine serum (Invitrogen), 2 mM L-glutamine (Invitrogen), and 2.5 µg/ml Plasmocin (InvivoGen) at 37°C in 5% CO_2_ humidified incubators. Primary fibroblast cell lines from two patients with SMA type I (GM03813 and GM03815) and one heterozygous carrier (GM03814, clinically unaffected mother of GM03813 and GM03815) were obtained from Coriell Cell Repository and maintained in MEM supplemented with 10% fetal bovine serum. The GM03815 cells were initially described in the Coriell Cell Repository as derived from a heterozygous carrier (clinically unaffected father of GM03813). However, snRNP deficiency and genetic linkage analysis of these cells showed that they in fact are derived from a homozygous carrier with SMA type I (a male sibling of GM03813) [34].

### RNA Preparations and Real-Time Quantitative Reverse Transcription PCR

Total RNA was extracted from tumor cell lines using the Trizol reagent (Invitrogen). The RNA was reverse transcribed with First-Strand cDNA synthesis using Superscript II (Invitrogen). Quantitative real-time PCR was carried out in the Applied Biosystems 7500 Real-Time PCR using transcript-specific TaqMan Gene Expression Assays (Applied Biosystems). The following probes were used: Hs01126636_g1 for detection of WRAP53 and Hs00167441_m1 for detection of ALAS1 as endogenous control.

### IF Microscopy

For IF experiments, cells were grown on sterilized cover slips, fixed with 100% MeOH at −20°C, permeabilized with 0.1% Triton X-100 for 5 min, and then blocked in blocking buffer (2% BSA, 5% glycerol, 0.2% Tween 20, and 0.1% NaN_3_). Cover slips were subsequently incubated for 1 h in primary antibody and 40 min in secondary antibody diluted in blocking buffer. The cover slips were mounted with Vectorshield mounting medium with DAPI (Vector Laboratories). Images were acquired with a Zeiss Axioplan 2 microscope, equipped with an AxioCam HRm camera using 43× or 60× oil immersion lenses, and processed using Axiovision Release 4.7.

### IPs and WB

For IP, cells were lysed in NP40 buffer (150 mM NaCl, 50 mM Tris-HCL [pH 8.0], 1% NP40, 1% PMSF, and 1% protease inhibitor cocktail) for 15 min on ice, followed by sonication 2× for 10 s. Extracts were spun down at 6,000 rpm for 5 min at 4°C and then quantified by Bradford assay (Bio-Rad). Endogenous proteins were immunoprecipitated with 1 µg of affinity-purified antibody per 1 mg extract supplemented with 10 µl of Dynabeads Protein G (Invitrogen) overnight at 4°C. The beads were washed 4× for 15 min with 1 ml of NP40 buffer and prepared for WB.

Cell extracts for WB analysis were prepared as previously described [1]. WB was performed according to standard procedures.

### Cell Fractionations

Cell fractionations were performed using a nuclear extract kit according to the manufacturer's instructions (Nuclear Extract Kit, Active Motif). For fractionation followed by IP, cells were lysed in hypotonic buffer (10 mM NaCl, 20 mM Tris-HCL [pH 7.5], 1% PMSF, and 1% protease inhibitor cocktail) for 20 min on ice. Samples were spun down at 3,600 rpm for 10 min at 4°C, and the supernatant (cytoplasmic fraction) was converted into NP40 buffer. The remaining pellet was lysed in NP40 buffer. The IP was performed as described earlier. In Figure 1B, equal volumes of each sample were loaded on an SDS-PAGE gel, whereas in Figure 3A, the maximal volume of each sample was loaded on the gel.

### Antibodies

Four different WRAP53 antibodies were used: rabbit α-WRAP53-C1 [1] (used for IP and WB), rabbit α-WRAP53-C2 (used for WB, IP, and IF), rabbit α-WRAP53 (Wdr79, A301-442A-1, Bethyl Laboratories; used for WB, IP, IF, and in situ PLA), and mouse polyclonal α-WRAP53 full-length (H00055135-B01, Abnova; used only in Figure S3A and S3B). To generate α-WRAP53-C2, rabbits were immunized with a KLH-conjugated WRAP53 peptide that maps to a region between aa 498–548 of full-length WRAP53 protein (Innovagen AB). The following antibodies were used in IF, IP, and WB: mouse α-coilin (ab11822, Abcam), rabbit α-coilin (sc-32860, Santa Cruz Biotechnology), mouse α-SMN (610647, BD Biosciences), mouse α-SMN (sc-32313, Santa Cruz Biotechnology), rabbit α-SMN (sc-15320, Santa Cruz Biotechnology), mouse α-Gemin3 (ab10305, Abcam), mouse α-Gemin3 (sc-57007, Santa Cruz Biotechnology), mouse α-Gemin2 (sc-57006, Santa Cruz Biotechnology), mouse α-importinβ (035K4852, Sigma), mouse α-importinβ (sc-137016, Santa Cruz Biotechnology), rabbit α-fibrillarin (ab5821, Abcam), mouse α-Sm (ab3138, Abcam), mouse α-SmB/B′ (sc-271094, Santa Cruz Biotechnology), mouse α-Hsp90α/β (sc-13119, Santa Cruz Biotechnology), rabbit α-Lamin A/C (sc-20681, Santa Cruz Biotechnology), rabbit IgG (sc-2027, Santa Cruz Biotechnology), mouse IgG (sc-2025, Santa Cruz Biotechnology), mouse α-Flag M2 (200472-21, Stratagene), rabbit α-GFP (ab290, Abcam), and mouse α-β-actin (Sigma). The following secondary antibodies were used: sheep α-mouse HRP (NA931V, GE Healthcare), donkey α-rabbit HRP (NA934V, GE Healthcare), swine α-rabbit FITC (F0054, Dako Cytomation), and horse α-mouse Texas Red (TI-2000, Vector).

### siRNA Oligonucleotides, Plasmids, and Transfection

The following siRNAs from Qiagen were used: siWRAP53#1 (SI00388941), siWRAP53#2 (SI00388948), siSMN1#7 (SI03108084), siSMN1#11 (SI04950932), siSMN1#12 (SI04950939), siCoilin#3 (SI00350343), siCoilin#7 (SI04330830), and a control siRNA (1027280). siRNA (10–20 nM) was transfected into cells using either Oligofectamine (Invitrogen) or HiPerfect (Qiagen) transfection reagents, in accordance with the supplier's recommendations. Flag-WRAP53 was cloned as described in Mahmoudi et al. [1]. To generate EGFP-tagged WRAP53 constructs, full-length or deletion mutants were amplified by PCR (Advantage-HF 2 polymerase, Clontech) and subcloned into pEGFP-C1 vector (Clontech). Flag-SMN was cloned by PCR (Advantage-HF 2 polymerase, Clontech) from SMN cDNA and subcloned into pCMV-Tag2 vector (Invitrogen). All primers used for PCR amplifications are listed in Table S1. Plasmid transfections were performed using Lipofectamine 2000 Reagent (Invitrogen).

For the generation of cells with stable overexpression of WRAP53, Flag-tagged full-length WRAP53 cDNA was cloned into the pLenti6/V5-D-TOPO vector (Invitrogen). The pLenti6-Flag-WRAP53 plasmid along with the pMDLg/RRE, pCMV-VSVG, and pRSV-Rev plasmids required for viral production were transfected into HEK293FT cells using Lipofectamine 2000 (Invitrogen). U20S cells were infected with viruses containing either pLenti6-Flag-WRAP53 or empty pLenti6/V5-D-TOPO vector, and positive cells were selected 48 h after infection using 10 µg/ml Blasticidin (Invitrogen).

### Statistical Analysis

All analyses were performed using Microsoft Office Excel 2003. Two-tailed Student's *t* test was used to determine statistical significance.

### In Situ PLA

In situ PLA experiments were performed as described previously [35]. Incubation with primary antibodies (0.4 ng/µl rabbit α-WRAP53 and 1 ng/µl mouse α-SMN in blocking solution) was performed at room temperature for 1 h. Cells were washed 3× for 5 min in PBS plus 0.1% Tween 20, with the first wash at 37°C. Secondary proximity probes (Rabbit-PLUS and Mouse-MINUS, Duolink kit, Olink Biosciences AB) were incubated for 2 h at 37°C. Cells were washed 1× for 5 min in 10 mM Tris-HCl (pH 7.5) plus 0.1% Tween 20 at 37°C, then 2× for 5 min in PBS plus 0.1% Tween 20. All subsequent steps were done according to the Duolink kit protocol (Olink Biosciences AB). FITC-labeled donkey α-rabbit F(ab′)_2_ fragment (Jackson ImmunoResearch) was added in order to counterstain for WRAP53. Images were acquired using an epifluorescent microscope (Axioplan 2, Zeiss) equipped with a 100-W mercury lamp, a CCD camera (C4742-95, Hamamatsu), emission filters for visualization of DAPI, FITC, and Cy3.5, and a 63× objective (plan-neofluar). WRAP53 staining was used to select image position.

## Supporting Information

Figure S1 **WRAP53 and coilin, but not SMN, are essential for Cajal body integrity.** (A) Quantitative real-time PCR analysis of U2OS cells treated with the indicated siRNAs for 48 h. (B) WB analysis of WRAP53, coilin, and SMN levels in U2OS cells treated with the indicated siRNA oligos for 48 h. β-actin was used as loading control. (C) IF staining of SMN, coilin, and WRAP53 in HeLa cells treated with siControl and siWRAP53#1 oligos for 48 h. Nuclei were stained with DAPI in all IF experiments. (D) Immunostainings of U2OS cells treated with the indicated siRNA oligos for 48 h, followed by staining with fibrillarin, SMN, or PML. Arrows indicate nucleoli in the fibrillarin staining and a gem in the SMN staining. (E) IF staining of WRAP53, SMN, and coilin in U2OS and HeLa cells treated with siControl and siCoilin#7 oligos for 48 h. (F) IF staining of WRAP53, coilin, and SMN in U2OS and HeLa cells treated with siControl, siSMN1#7, or siSMN1#11 oligos for 48 h.
(3.15 MB TIF)

Figure S2 **High exogenous expression of WRAP53 disrupts Cajal body structure.** (A) Immunostaining of U2OS cells transiently transfected with Flag-WRAP53 for 16 h, followed by staining with Flag- and WRAP53-specific antibodies. (B) U2OS cells transiently transfected with EGFP-tagged full-length WRAP53 for 16 h and stained for coilin or SMN. (C) WB analysis of WRAP53, coilin, and SMN levels in U2OS cells overexpressing Flag-tagged WRAP53. β-actin was used as loading control. (D) IF staining of Flag and PML in U2OS cells transiently transfected with Flag-tagged WRAP53 for 16 h. Arrows indicate an untransfected cell.
(1.84 MB TIF)

Figure S3 **WRAP53 is expressed in the cytoplasm and in Cajal bodies.** (A) WB analysis of the four WRAP53 antibodies mentioned in the paper. Full-length filters are shown to demonstrate the specificity of the WRAP53 antibodies. The rabbit α-WRAP53 (Wdr79, A301-442A-1, Bethyl Laboratories) antibody was used in all IF stains shown in the main figures. This is also the same antibody employed by Tycowski et al. [2],[3]. The mouse polyclonal α-WRAP53 full-length antibody (H00055135-B01, Abnova) corresponds to the anti-TCAB1 antibody used by Venteicher et al. [2],[3]. (B) IF staining of endogenous WRAP53 in U2OS cells using α-WRAP53-C2 (top) and α-WRAP53 full-length (bottom) antibodies. (C) Immunostaining of endogenous WRAP53 using the α-WRAP53 (Wdr79) antibody and FA fixation protocol in U2OS cells. Shortly, cells were grown on sterilized cover slips and fixed with 4% FA for 10 min at room temperature. The cells were then permeabilized with 0.1% Triton X-100 for 3 min at room temperature, followed by 30 min of blocking in blocking buffer (2% BSA and 5% glycerol). Cover slips were subsequently incubated for 1 h in primary antibody and 40 min in secondary antibody diluted in blocking buffer. The cover slips were mounted with Vectorshield mounting medium with DAPI (Vector Laboratories).
(1.30 MB TIF)

Figure S4 **Stable Flag-WRAP53 cells with high WRAP53 expression lack Cajal bodies.** (A) IF analysis of U2OS cells stably transfected with either Flag–Empty vector (Mock cells) or Flag-WRAP53 (Flag-WRAP53 cells) stained for WRAP53 and coilin. (B) WRAP53 interacts with SMN both in the cytoplasm and in the nucleus. In situ PLA of WRAP53–SMN interaction in WRAP53-depleted U2OS cells as negative control. A clear reduction in in situ PLA signals was observed in siWRAP53 compared to siControl, confirming the specificity of detection in Figure 5E. Co-IF with WRAP53 is shown in green, and the nuclear staining in blue.
(1.34 MB TIF)

Figure S5 **Aberrant expression of WRAP53 leads to mislocalization of Gemin3 and Gemin2.** (A) WB analysis of WRAP53 and SMN levels in fractionated U2OS cells treated with the indicated siRNA oligos for 48 h. Lamin A/C and Hsp90 were used as nuclear and cytoplasmic markers, respectively. (B) IP of endogenous WRAP53 from U2OS cells pretreated with the indicated siRNA oligos for 48 h. (C) IP of endogenous SMN from U2OS cells pretreated with the indicated siRNA oligos for 48 h. (D) IF of U2OS cells treated with the indicated siRNA oligos for 48 h, followed by staining with Gemin3- (ab10305, Abcam) and WRAP53-specific antibodies. Arrows indicate a gem. (E) IF staining of WRAP53 and Gemin3 (ab10305, Abcam) in U2OS cells transiently transfected with Flag-tagged WRAP53 for 16 h. Arrows indicate an untransfected cell. (F) IF of U2OS cells treated with the indicated siRNA oligos for 48 h, followed by staining with SMN-, Gemin2-, Gemin3- (sc-57007, Santa Cruz Biotechnology), and WRAP53-specific antibodies.
(3.60 MB TIF)

Figure S6 **WRAP53-depleted cells show altered localization of Sm but no change in intracellular distribution of Sm.** (A) IF of U2OS cells treated with the indicated siRNA oligos for 48 h, followed by staining with Sm-, coilin-, and WRAP53-specific antibodies. (B) WB analysis of Sm levels in fractionated U2OS cells treated with the indicated siRNA oligos.
(1.07 MB TIF)

Table S1 **PCR primers for cloning of EGFP-WRAP53 and Flag-SMN constructs.**
(0.71 MB TIF)

## Abbreviations

aa: amino acids
EGFP: enhanced green fluorescent protein
GFP: green fluorescent protein
IF: immunoflourescence
in situ PLA: in situ proximity ligation assay
IP: immunopreciptation
PML: promyelocytic leukemia
RNP: ribonucleoprotein
scaRNA: small Cajal body–specific RNA
siRNA: small interfering RNA
SMA: spinal muscular atrophy
SMN: survival of motor neuron
snRNP: small nuclear ribonucleoprotein WB, Western blotting
