# Correction: WRAP53 Is Essential for Cajal Body Formation and for Targeting the Survival of Motor Neuron Complex to Cajal Bodies

**DOI:** 10.1371/journal.pbio.3000030

**Published:** 2018-09-25

**Authors:** Salah Mahmoudi, Sofia Henriksson, Irene Weibrecht, Stephen Smith, Ola Söderberg, Staffan Strömblad, Klas G. Wiman, Marianne Farnebo

Fig 3 is incorrect. The authors have provided a corrected version here. The left panel of Fig 3A has been replaced with a replicate. This panel was accidentally swapped and the data for IB: WRAP53 was reused in Fig 6C. The legend remains the same.

**Fig 3:**
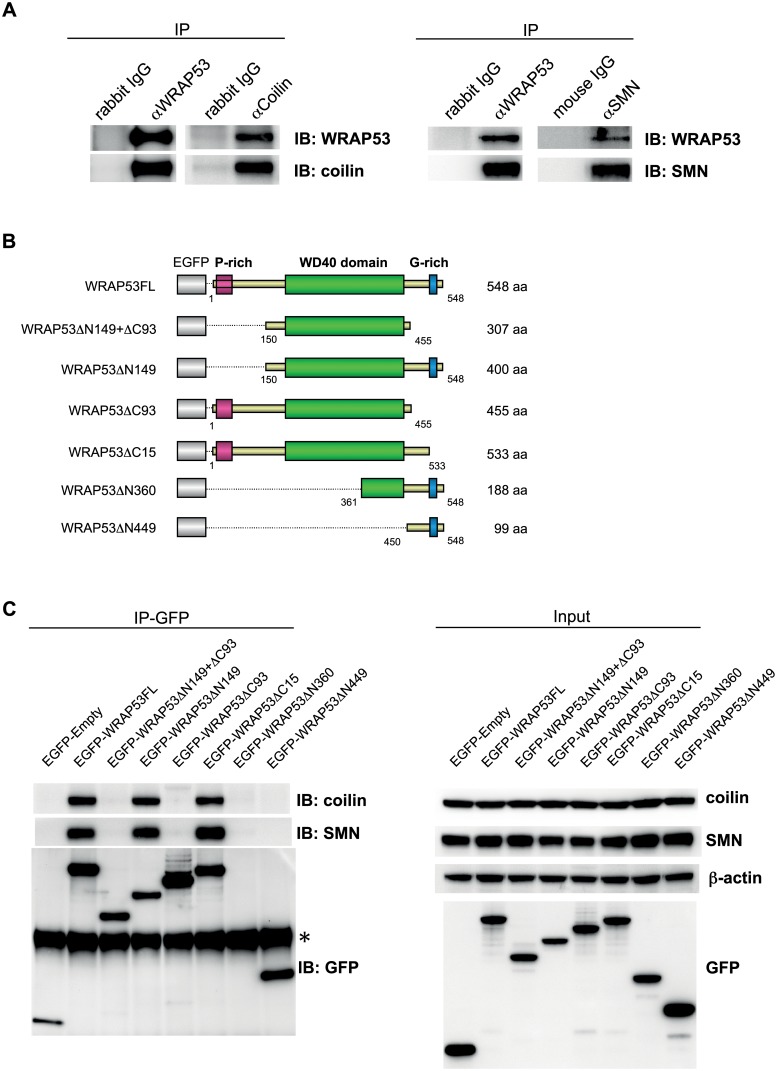
WRAP53 binds coilin and SMN via its WD40 domain and C-terminal region. (A) IP of endogenous WRAP53, coilin, and SMN from U2OS cells followed by immunoblotting (IB) with the indicated antibodies. Rabbit and mouse IgG were used as negative controls. (B) Schematic illustration of EGFP-tagged WRAP53 deletion constructs: WRAP53FL (full-length), WRAP53ΔN149+ΔC93 (contains aa 150–455), WRAP53ΔN149 (contains aa 150–584), WRAP53ΔC93 (contains aa 1–455), WRAP53ΔC15 (contains aa 1–533), WRAP53ΔN360 (contains aa 361–548), and WRAP53ΔN449 (contains aa 450–548). The EGFP protein has a molecular weight of approximately 27 kDa. (C) U2OS cells transfected with the indicated WRAP53 constructs for 16 h, followed by IP with GFP antibody. Asterisk indicates the heavy chain. The WRAP53ΔN360 product is 50 kDa in size and is thus covered by the heavy chain.

Fig 5 is incorrect. The authors have provided a corrected version here. A splice-line has been included to clearly indicate the lanes in Fig 5D. The legend remains the same.

**Fig 5:**
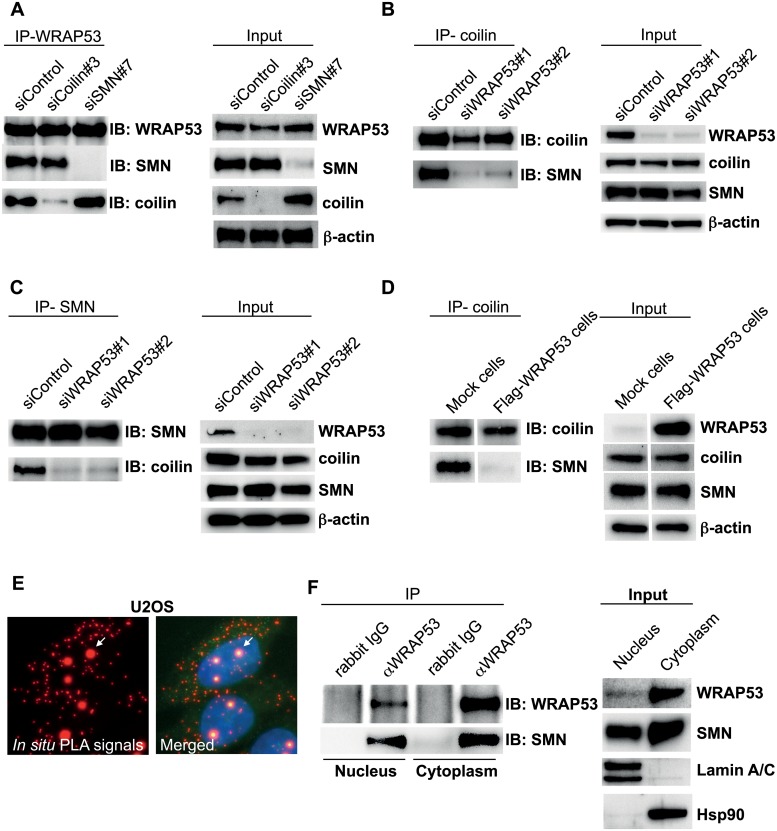
WRAP53 mediates coilin–SMN interaction and associates with SMN both in the cytoplasm and in the nucleus. (A–C) IP of endogenous WRAP53 (A), coilin (B), and SMN (C) from U2OS cells pretreated with the indicated siRNA oligos for 48 h. (D) IP of endogenous coilin from U2OS cells stably transfected with empty vector (Mock cells) or Flag-WRAP53 (Flag-WRAP53 cells). The Flag-WRAP53 cells express high levels of WRAP53 and show subsequent disruption of Cajal bodies (see Figure S4A). (E) In situ PLA detection of the interaction between WRAP53 and SMN in U2OS cells. Protein–protein interactions are visualized as small, distinct red spots. In Cajal bodies several in situ PLA signals are superimposed. Arrows indicate one Cajal body. (F) IP of endogenous WRAP53 from nuclear and cytoplasmic fractions of U2OS cells.

Fig 7 is incorrect. The authors have provided a corrected version here. The lower left image (IB: coilin) of Fig 7C has been flipped as it was originally presented upside down. A new legend is provided.

**Fig 7:**
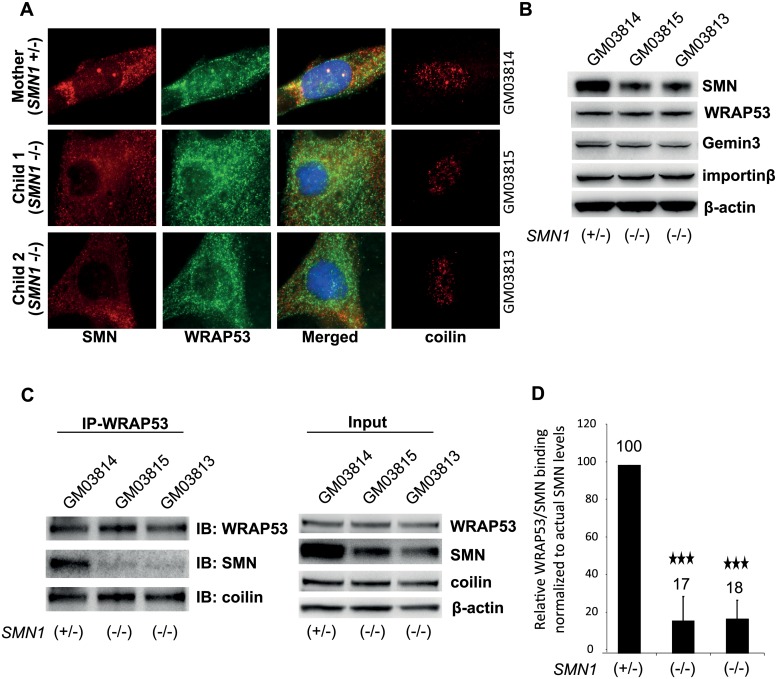
Interaction of WRAP53 and SMN is disrupted in SMA type I patients. (A) IF staining of SMN, WRAP53, and coilin in fibroblasts derived from the unaffected mother (GM03814) and her two children with SMA type I (GM03815 and GM03813). (B) WB analysis of WRAP53, SMN, Gemin3, and importinβ levels in fibroblasts from the same individuals as described in (A). β-actin was used as loading control. (C) IP of endogenous WRAP53 from the same fibroblasts described in (A) Uncropped images of the original gels are shown in S7 Fig. (D) Densitometric quantifications of the relative WRAP53–SMN interaction in the fibroblasts described in (A). Levels of SMN co-immunoprecipitated with WRAP53 antibody have been normalized to the levels of immunoprecipitated WRAP53, as well as to the relative amount of SMN in the cells. The latter was calculated by dividing SMN levels in the input with β-actin levels in the input. The graph shows means of three independent experiments; error bars represent standard error, three asterisks indicate p<0.001 (Student’s t test).

An additional supplementary file, named S7 Fig, shows the uncropped data for Fig 7C.

## Supporting information

S7 Fig Uncropped images of the immunoblots displayed in Fig 7C. To allow for simultaneous detection of proteins of different molecular weights, membranes were at times cut horizontally approximately 25 kD below and above the expected molecular weight, prior to incubation with antibodies. In the instances the proteins had similar molecular weights (as for coilin and WRAP53) two parallel membranes were blotted and analyzed. However, for IP experiments that test the binding between immunoprecipitated and co-precipitated proteins during different conditions, blotting is required on the same filter to ensure that the loading does not impact the interpretation of the results. In such cases, antibodies that were raised in different species were used (rabbit for WRAP53 and mouse for coilin) to minimize the risk of cross-reaction, and the co-precipitated protein was always blotted first, followed by detection of the immunoprecipitated protein itself.
(EPS)
