# Supplementary figures and images for: WRAP53 Is Essential for Cajal Body Formation and for Targeting the Survival of Motor Neuron Complex to Cajal Bodies
Source: PLoS Biol. 2010 Nov 2;8(11):e1000521. doi: 10.1371/journal.pbio.1000521 (PMC2970535; doi:10.1371/journal.pbio.1000521)

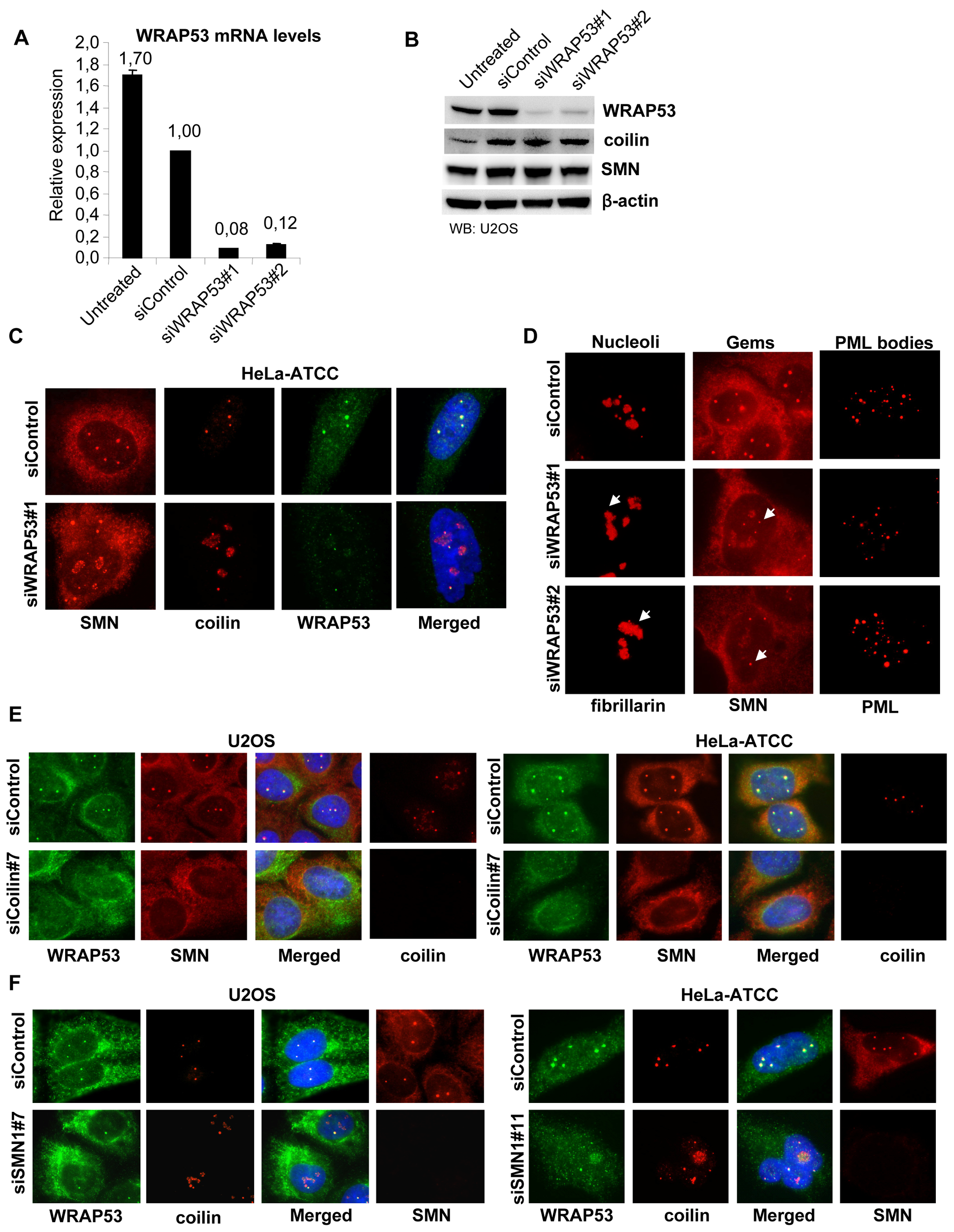

Supplement: Figure S1 — WRAP53 and coilin, but not SMN, are essential for Cajal body integrity. (A) Quantitative real-time PCR analysis of U2OS cells treated with the indicated siRNAs for 48 h. (B) WB analysis of WRAP53, coilin, and SMN levels in U2OS cells treated with the indicated siRNA oligos for 48 h. β-actin was used as loading control. (C) IF staining of SMN, coilin, and WRAP53 in HeLa cells treated with siControl and siWRAP53#1 oligos for 48 h. Nuclei were stained with DAPI in all IF experiments. (D) Immunostainings of U2OS cells treated with the indicated siRNA oligos for 48 h, followed by staining with fibrillarin, SMN, or PML. Arrows indicate nucleoli in the fibrillarin staining and a gem in the SMN staining. (E) IF staining of WRAP53, SMN, and coilin in U2OS and HeLa cells treated with siControl and siCoilin#7 oligos for 48 h. (F) IF staining of WRAP53, coilin, and SMN in U2OS and HeLa cells treated with siControl, siSMN1#7, or siSMN1#11 oligos for 48 h. (3.15 MB TIF) [file pbio.1000521.s001.tif]

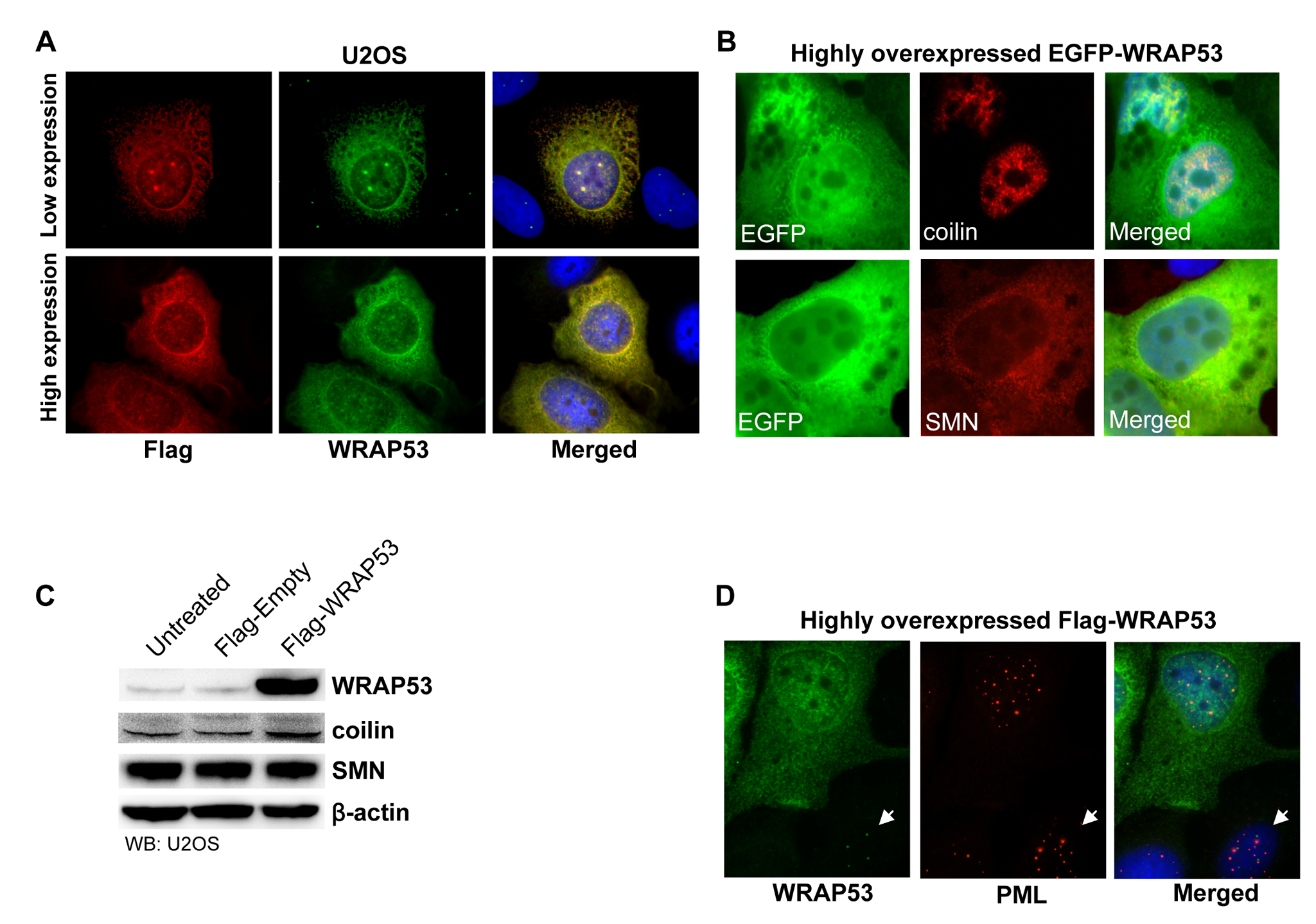

Supplement: Figure S2 — High exogenous expression of WRAP53 disrupts Cajal body structure. (A) Immunostaining of U2OS cells transiently transfected with Flag-WRAP53 for 16 h, followed by staining with Flag- and WRAP53-specific antibodies. (B) U2OS cells transiently transfected with EGFP-tagged full-length WRAP53 for 16 h and stained for coilin or SMN. (C) WB analysis of WRAP53, coilin, and SMN levels in U2OS cells overexpressing Flag-tagged WRAP53. β-actin was used as loading control. (D) IF staining of Flag and PML in U2OS cells transiently transfected with Flag-tagged WRAP53 for 16 h. Arrows indicate an untransfected cell. (1.84 MB TIF) [file pbio.1000521.s002.tif]

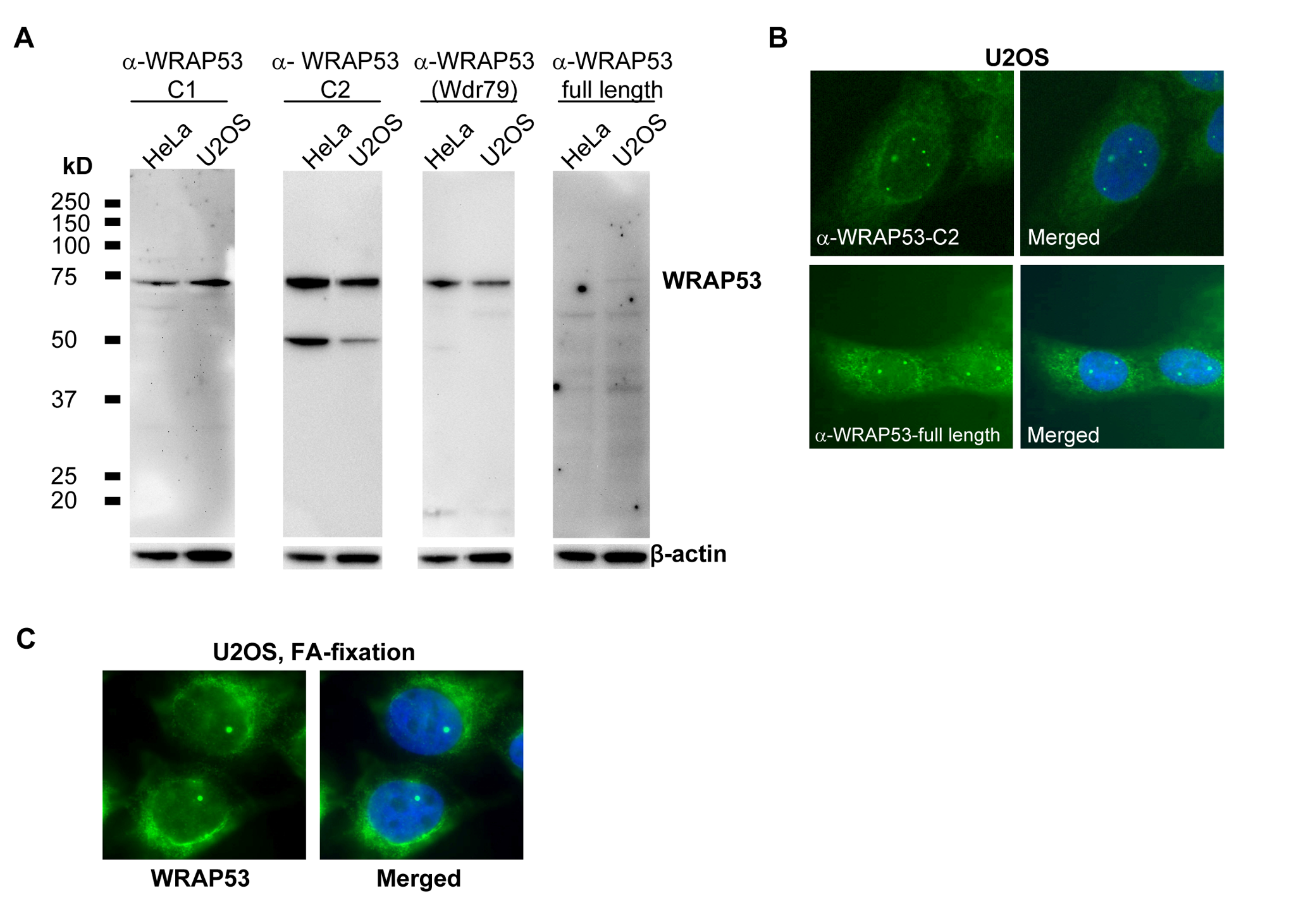

Supplement: Figure S3 — WRAP53 is expressed in the cytoplasm and in Cajal bodies. (A) WB analysis of the four WRAP53 antibodies mentioned in the paper. Full-length filters are shown to demonstrate the specificity of the WRAP53 antibodies. The rabbit α-WRAP53 (Wdr79, A301-442A-1, Bethyl Laboratories) antibody was used in all IF stains shown in the main figures. This is also the same antibody employed by Tycowski et al. [2],[3]. The mouse polyclonal α-WRAP53 full-length antibody (H00055135-B01, Abnova) corresponds to the anti-TCAB1 antibody used by Venteicher et al. [2],[3]. (B) IF staining of endogenous WRAP53 in U2OS cells using α-WRAP53-C2 (top) and α-WRAP53 full-length (bottom) antibodies. (C) Immunostaining of endogenous WRAP53 using the α-WRAP53 (Wdr79) antibody and FA fixation protocol in U2OS cells. Shortly, cells were grown on sterilized cover slips and fixed with 4% FA for 10 min at room temperature. The cells were then permeabilized with 0.1% Triton X-100 for 3 min at room temperature, followed by 30 min of blocking in blocking buffer (2% BSA and 5% glycerol). Cover slips were subsequently incubated for 1 h in primary antibody and 40 min in secondary antibody diluted in blocking buffer. The cover slips were mounted with Vectorshield mounting medium with DAPI (Vector Laboratories). (1.30 MB TIF) [file pbio.1000521.s003.tif]

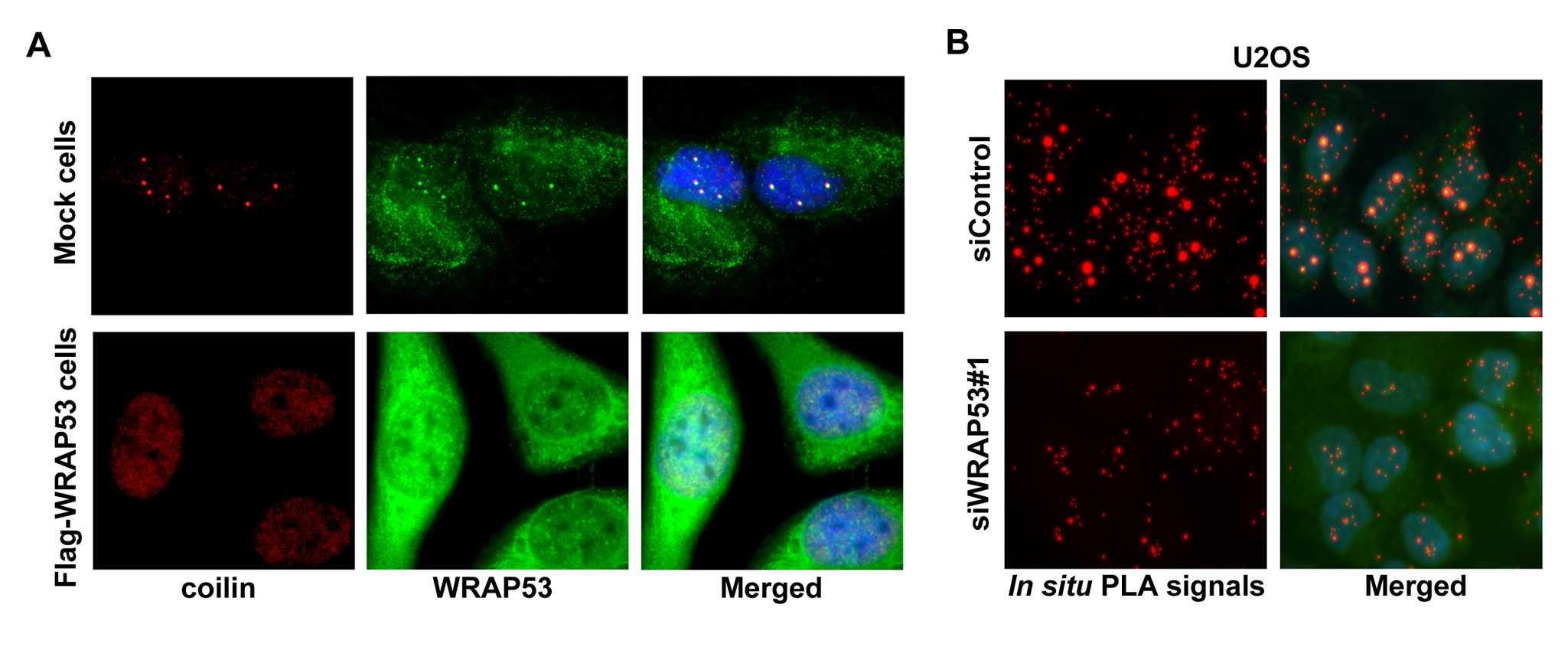

Supplement: Figure S4 — Stable Flag-WRAP53 cells with high WRAP53 expression lack Cajal bodies. (A) IF analysis of U2OS cells stably transfected with either Flag–Empty vector (Mock cells) or Flag-WRAP53 (Flag-WRAP53 cells) stained for WRAP53 and coilin. (B) WRAP53 interacts with SMN both in the cytoplasm and in the nucleus. In situ PLA of WRAP53–SMN interaction in WRAP53-depleted U2OS cells as negative control. A clear reduction in in situ PLA signals was observed in siWRAP53 compared to siControl, confirming the specificity of detection in Figure 5E. Co-IF with WRAP53 is shown in green, and the nuclear staining in blue. (1.34 MB TIF) [file pbio.1000521.s004.tif]

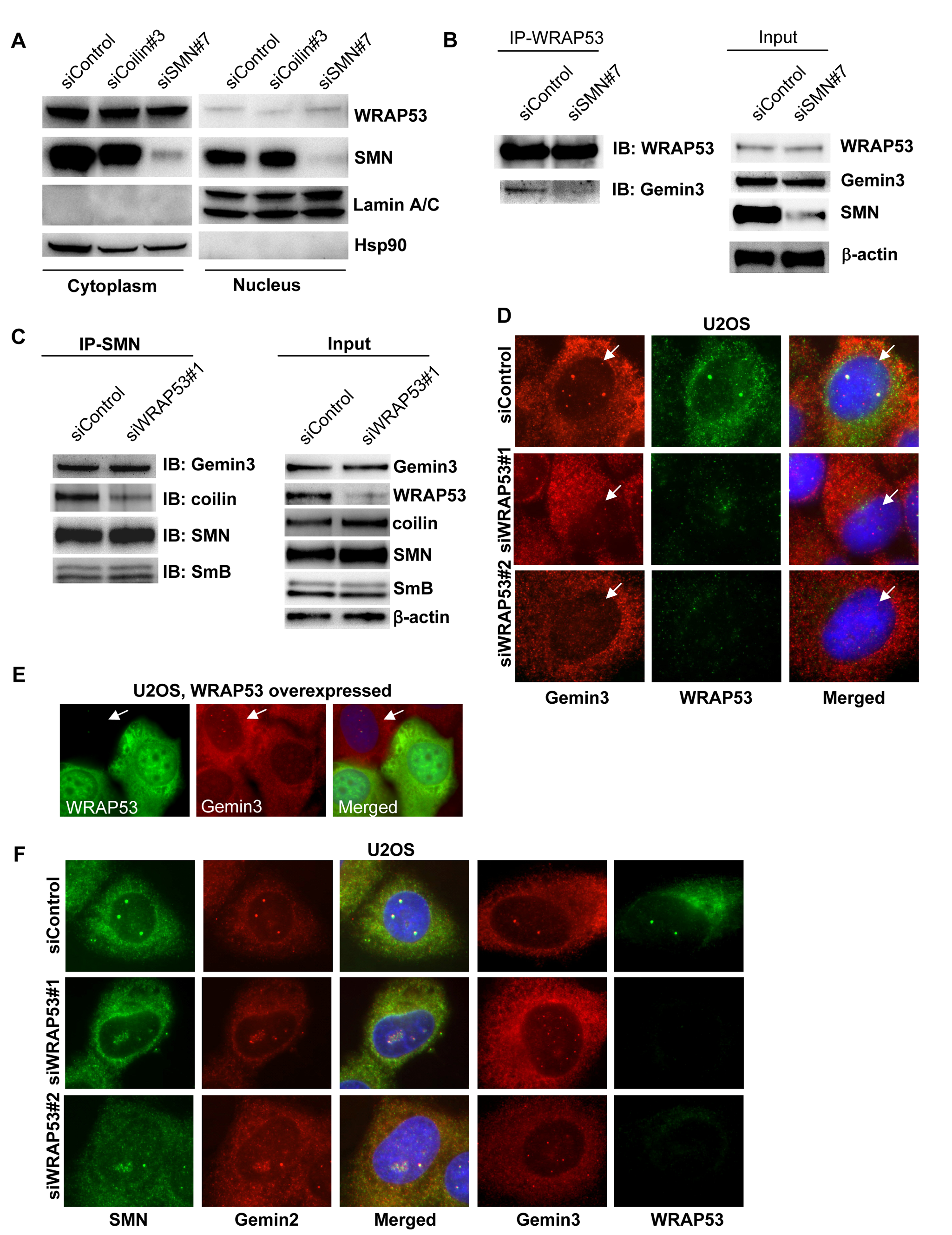

Supplement: Figure S5 — Aberrant expression of WRAP53 leads to mislocalization of Gemin3 and Gemin2. (A) WB analysis of WRAP53 and SMN levels in fractionated U2OS cells treated with the indicated siRNA oligos for 48 h. Lamin A/C and Hsp90 were used as nuclear and cytoplasmic markers, respectively. (B) IP of endogenous WRAP53 from U2OS cells pretreated with the indicated siRNA oligos for 48 h. (C) IP of endogenous SMN from U2OS cells pretreated with the indicated siRNA oligos for 48 h. (D) IF of U2OS cells treated with the indicated siRNA oligos for 48 h, followed by staining with Gemin3- (ab10305, Abcam) and WRAP53-specific antibodies. Arrows indicate a gem. (E) IF staining of WRAP53 and Gemin3 (ab10305, Abcam) in U2OS cells transiently transfected with Flag-tagged WRAP53 for 16 h. Arrows indicate an untransfected cell. (F) IF of U2OS cells treated with the indicated siRNA oligos for 48 h, followed by staining with SMN-, Gemin2-, Gemin3- (sc-57007, Santa Cruz Biotechnology), and WRAP53-specific antibodies. (3.60 MB TIF) [file pbio.1000521.s005.tif]

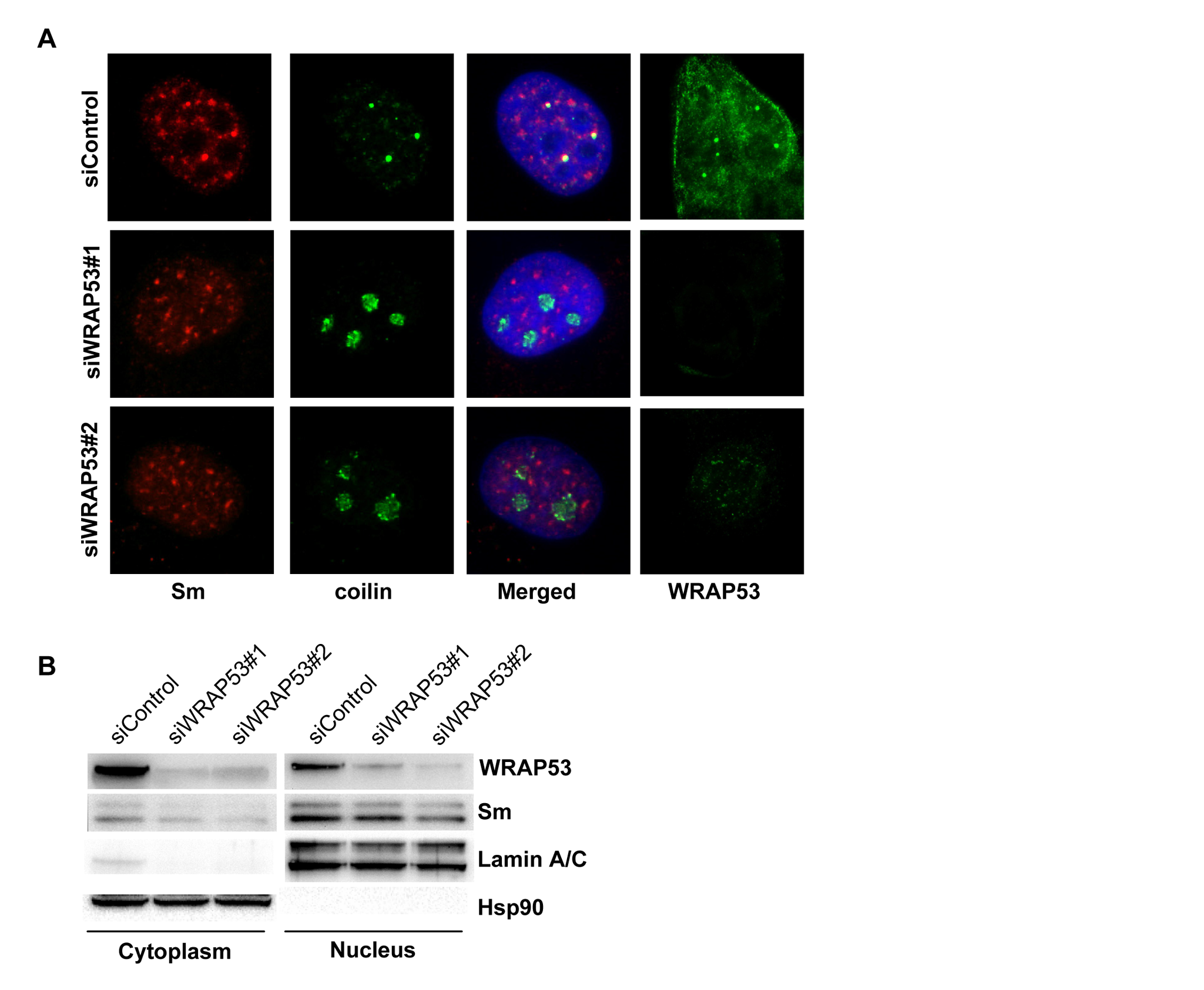

Supplement: Figure S6 — WRAP53-depleted cells show altered localization of Sm but no change in intracellular distribution of Sm. (A) IF of U2OS cells treated with the indicated siRNA oligos for 48 h, followed by staining with Sm-, coilin-, and WRAP53-specific antibodies. (B) WB analysis of Sm levels in fractionated U2OS cells treated with the indicated siRNA oligos. (1.07 MB TIF) [file pbio.1000521.s006.tif]

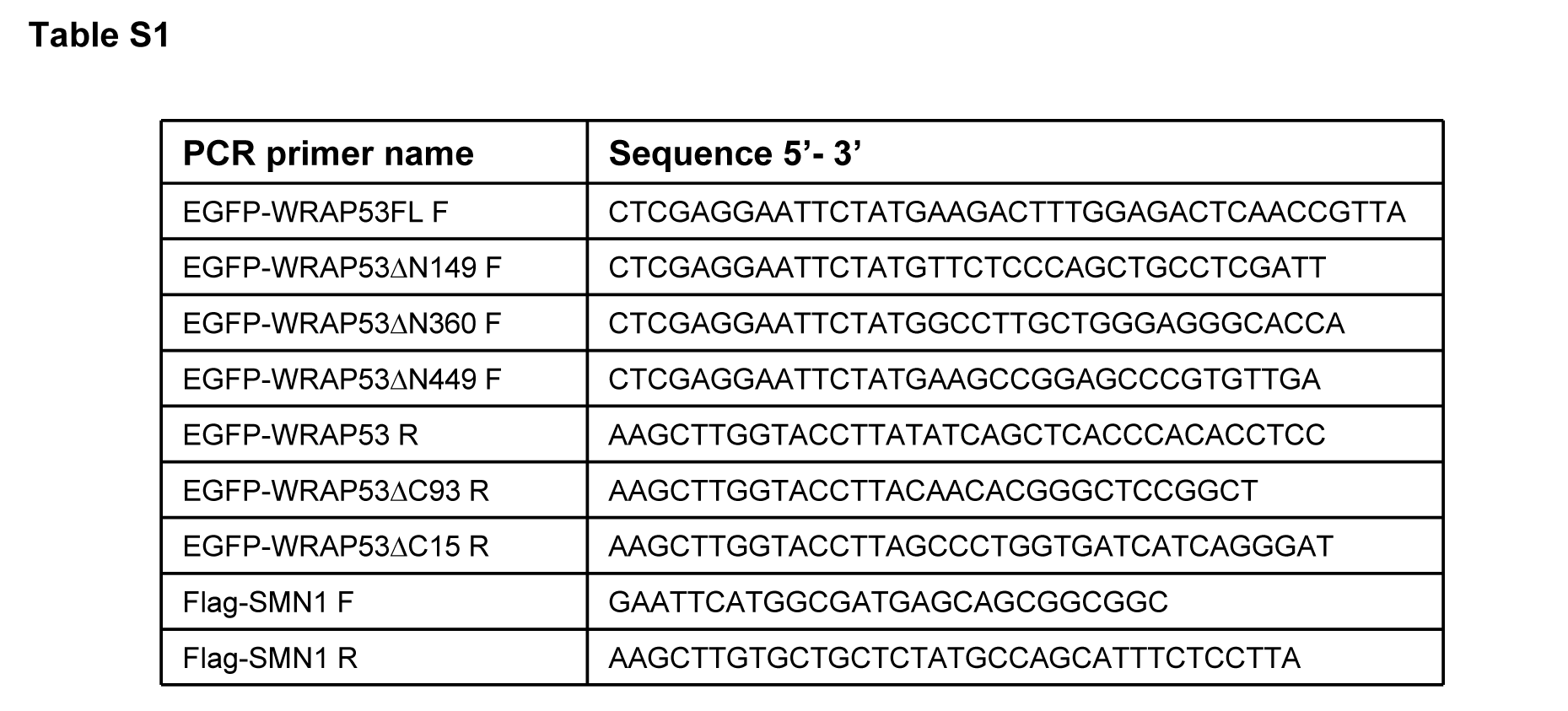

Supplement: Table S1 — PCR primers for cloning of EGFP-WRAP53 and Flag-SMN constructs. (0.71 MB TIF) [file pbio.1000521.s007.tif]
